# Causal effects of physical activity and screen time on childhood intelligence via Mendelian randomization: The mediating role of intracranial volume

**DOI:** 10.1016/j.dcn.2025.101586

**Published:** 2025-06-20

**Authors:** Junjiao Feng, Yi Wan, Liang Zhang

**Affiliations:** a Key Research Base of Humanities and Social Sciences of the Ministry of Education, Academy of Psychology and Behavior, Tianjin Normal University, Tianjin 300387, China; b Faculty of Psychology, Tianjin Normal University, Tianjin 300387, China; c Tianjin Key Laboratory of Student Mental Health and Intelligence Assessment, Tianjin 300387, China; d Department of Psychology, Institute of Education, China West Normal University, Nanchong 637002, China

**Keywords:** Leisure Screen time, Moderate to Vigorous Physical Activity, Intracranial Volume, Childhood IQ, Mendelian randomization

## Abstract

Growing evidence suggests that physical activity and screen time affect intelligence (IQ) during childhood, a critical period for brain development, yet the relationship between these factors remains controversial. Using bidirectional Mendelian randomization (MR), we investigated these associations while accounting for potential reverse causality. Our two-sample MR analysis revealed a positive causal effect of moderate to vigorous physical activity (PA) on childhood IQ (*β* = 0.42, 95 % confidence interval (CI): [0.12, 0.72], *p* = 6.26 × 10^−3^), whereas leisure screen time (LST) exhibited a negative causal effect (*β* = −0.35, 95 % CI: [ −0.60, −0.10], *p* = 5.59 ×10^−3^). Reverse MR analysis found no evidence of causations. A two-step MR mediation framework further suggested that the intracranial volume (ICV) mediated 21.69 % (95 % CI: [15.25 %, 28.13 %]) of the negative effect of LST on childhood IQ. These MR-derived findings demonstrate that PA positively influences childhood IQ, whereas LST negatively impacts it, partly through reduced ICV. By leveraging genetic instruments, this study strengthens causal inference and highlights the potential of PA promotion and screen time reduction to support cognitive development. Further research is needed to elucidate the mechanisms underlying these associations and their long-term cognitive consequences.

## 1 Introduction

Intelligence, commonly indexed by intelligence quotient (IQ), reflects the capacity to learn from experience and to adapt to, shape, and select environments (Sternberg, 2012). Contemporary conceptualizations of intelligence trace back to Spearman's seminal work, which proposed a general cognitive factor (g) underlying performance across diverse intellectual tasks, alongside task-specific abilities (Spearman, 1928). Empirical investigations spanning nearly a century have consistently identified this general factor across various test batteries. IQ is a robust predictor of academic achievement (Flores-Mendoza et al., 2021, Gustafsson and Balke, 1993, Roth et al., 2015), occupational performance (Kuncel et al., 2004, Ree et al., 1994), and health outcomes, including morbidity and longevity (Gottfredson and Deary, 2004). Identifying determinants of IQ during childhood is thus critical for informing interventions to optimize cognitive development.

Genome‑wide association studies (GWAS) indicate substantial heritability of childhood IQ (Sniekers et al., 2017), while environmental factors also exert a meaningful influence (Sternberg, 2012). Among modifiable behaviors, physical activity has emerged as a cost-effective intervention to enhance brain structure and cognitive function (Min et al., 2024, Stillman et al., 2020, Zou et al., 2024). For instance, Migueles et al. (2021) found that stable, non-fragmented physical activity patterns are associated with improved executive function and academic performance in children.

Concurrently, the proliferation of digital media has increased leisure screen time (LST) among youth, often displacing physical activity*.* Empirical evidence links prolonged LST to adverse brain outcomes, including reduced volume, altered connectivity, and impaired function (Hutton et al., 2020, Maza et al., 2023, Paulus et al., 2019). Moreover, elevated screen exposure associated with cognitive deficits such as increased impulsivity, reduced cognitive flexibility, and poorer decision-making in children and adolescents (Odgers and Jensen, 2020, Portugal et al., 2023, Sina et al., 2023, Stiglic and Viner, 2019).

Intracranial volume (ICV), an anatomical proxy for maximal brain size, serves as a biomarker of cognitive capacity (MacLullich et al., 2002). Larger ICV consistently correlates with superior fluid intelligence and executive function throughout the lifespan (MacLullich et al., 2002, Manfro et al., 2021, Roussotte et al., 2012, van Loenhoud et al., 2022).

Despite abundant observational associations among physical activity, LST, ICV, and IQ, causal inference remains limited. Mendelian randomization (MR) leverages genetic variants as instrumental variables (IVs) to disentangle causality from confounding (Emdin et al., 2017, Guo et al., 2025, Rees et al., 2020, Sun et al., 2024; Wang et al., 2024; Ziegler et al., 2015). While prior studies have examined physical activity or LST in isolation, and separate work has linked these behaviors to brain structural changes (Fox et al., 2022, Li et al., 2024, Paulus et al., 2019), no study to our knowledge has evaluated whether ICV mediates their causal effects on childhood IQ.

**Fig. 1:**
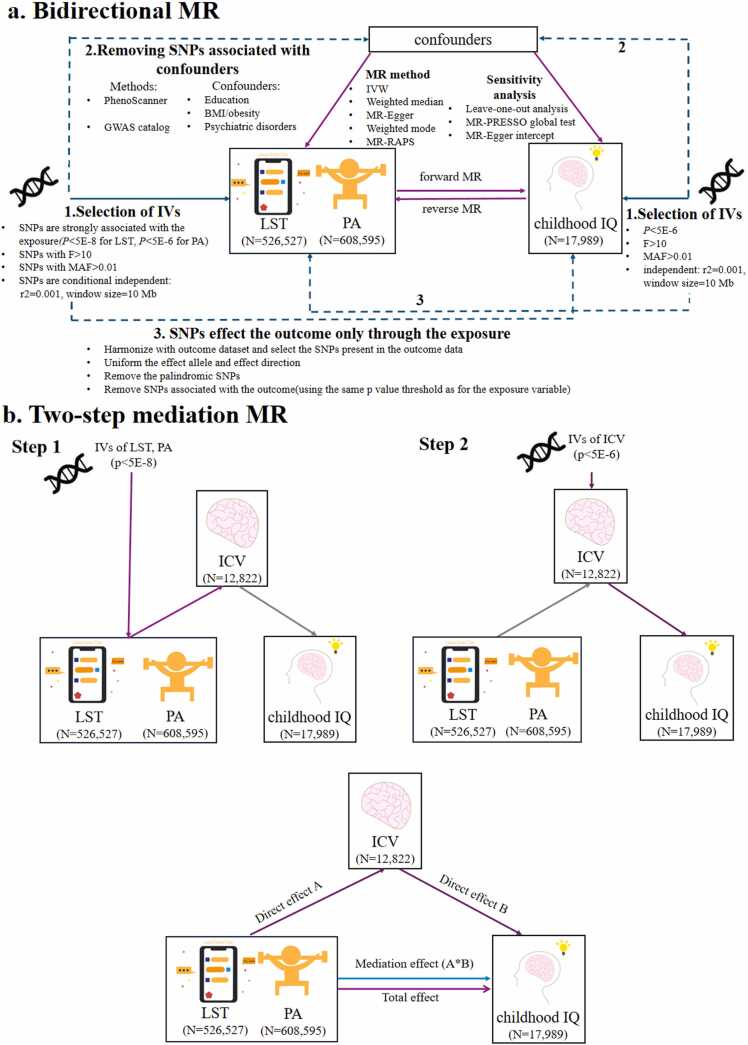
Flowchart of this study. **a**: A bidirectional two-sample MR analysis to investigate the causal effects of LST and PA on childhood IQ; **b**: A two-step MR analysis estimated the causal mediation effect of LST (or PA) on childhood IQ via ICV. LST = Leisure Screen Time; PA = Moderate-to-Vigorous Physical Activity; ICV = Intracranial Volume; IV = instrumental variables.

In this study, we aim to explore the causal relationships between LST (or physical activity), childhood IQ, and ICV, as well as the mediation pathways involved. Our study utilizes data from open genome-wide association studies (GWAS), including the GWAS of moderate to vigorous physical activity (PA) during leisure time (Wang et al., 2022), LST (Wang et al., 2022), ICV (Hibar et al., 2015), and childhood IQ (Benyamin et al., 2014). We first conducted a bidirectional two-sample MR analysis to assess the causal effects of LST and PA on childhood IQ. In the forward MR analysis, we estimated the causal effect of PA and LST on childhood IQ, while the reverse MR analysis focused on the effect of childhood IQ on PA and LST. Additionally, we examined the potential mediating role of ICV in the causal relationship between LST (or PA) and childhood IQ. To address these questions, we performed three analyses: the first investigated the causal effect of PA and LST on ICV, the second examined the causal effect of ICV on childhood IQ, and the third assessed the mediating effect of ICV on the causal pathways from LST (or PA) to childhood IQ (Fig. 1).

## 2 Methods

### 2.1 The design of the study

This study employed a two-sample Mendelian randomization (MR) method to investigate the causal effects of moderate to vigorous physical activity (PA) and leisure screen time (LST) on childhood Intelligence (IQ), while exploring potential mediating factors in these relationships. First, we conducted two-sample MR analyses to examine the causal effects of (1) PA on childhood IQ, (2) LST on childhood IQ, and (3) the potential for reverse causation. Second, we performed a two-step MR mediation analysis to evaluate whether intracranial volume (ICV) mediates the observed causal relationships between LST (or PA) and childhood IQ. The study flowchart is presented in Fig. 1.

### 2.2 Date source

#### 2.2.1 Genome-wide association studies (GWAS) summary data for PA and LST

We obtained GWAS summary statistics for PA and LST from the largest GWAS database (Wang et al., 2022). PA was defined as activities requiring ≥ 3 metabolic equivalents (METs) at least one hour per week, including moderate-intensity (e.g., light cycling) and vigorous-intensity activities (e.g., fast cycling, aerobics). PA measurements were derived from both self-reported questionnaires and wrist-worn accelerometer data. For self-reported data, moderate-intensity minutes were multiplied by 4 METs and vigorous-intensity minutes by 8 METs to calculate total activity. LST refers to the time spent using electronic devices like TVs, smartphones, tablets, or computers. It is measured through self-reported data, where participants provide information about the hours spent on activities such as watching TV, playing video games, or using a computer during their leisure time. To minimize potential confounding from population stratification, we restricted our MR analysis to individuals of European ancestry. Detailed cohort characteristics are provided in supplemental Table S1.

#### 2.2.2 GWAS summary data for childhood IQ

The GWAS data for childhood IQ were obtained from the CHIC (Childhood Intelligence Consortium) study (http://www.childhoodintelligence.org/). This consortium collected genome-wide SNP data and intelligence scores from 17,989 European ancestry children across six discovery cohorts and three replication cohorts (Benyamin et al., 2014). Detailed cohort characteristics are provided in supplemental Table S2.

#### 2.2.3 GWAS summary data for ICV

The summary-level GWAS data for ICV were derived from a large-scale meta-analysis conducted by the Enhancing Neuro Imaging Genetics through Meta-Analysis (ENIGMA) Consortium (Hibar et al., 2015). This study included 12,822 individuals of European ancestry across 28 independent cohorts. Detailed cohort characteristics are provided in supplemental Table S3.

To ensure that the samples used for exposures in the GWAS study were independent from those used for outcomes, we conducted a manual review of the sample descriptions for each GWAS study. For LST and PA, we derived genetic instruments from the largest GWAS meta-analysis to date (N = 703,901, 51 cohorts of European ancestry). For childhood IQ and ICV, summary statistics were sourced from the CHIC consortium and ENIGMA consortium, respectively, ensuring avoidance of sample overlap between exposures and outcomes in our MR analyses.

### 2.3 Selection of IVs

We employed a two-sample MR approach using summary-level data, where Single Nucleotide Polymorphisms (SNPs) served as instruments for the risk factor. MR analysis must satisfy the following three assumptions: (1) The genetic variants used as IVs must strongly associate with the exposure, otherwise, it may lead to bias due to weak instruments. This correlation strength is typically assessed with the F-statistic, where an F-statistic value above 10 is generally regarded as a low likelihood of bias from weak instruments; (2) The IVs should not be linked to any known confounders, meaning they should influence the outcome only through the exposure and not through other pathways. (3) The IVs must be unrelated to the outcome and should not have a direct effect on the outcome without passing through the exposure. To satisfy the first assumption, we selected two sets of *p* values for the genetic variants associated with the exposure in our MR analysis. We set the threshold of *p* < 5 × 10^−8^ as the standard for genome-wide significance to select IVs, however, when resulting in very few (usually less than 4) or even no SNPs, we relaxed the statistical threshold (*p* < 5 ×10^−6^) for selecting IVs, this method of relaxing the statistical threshold for IVs has been used in previous high quality MR studies (Choi et al., 2019). In particular, we used a threshold of *p* < 5 × 10^−8^ to select IVs for estimating the causal effects of LST on childhood IQ, *p* < 5 × 10^−6^ for the MR of estimating the causal effect of PA on childhood IQ. In the inverse MR analysis for causal estimation of childhood IQ on LST and PA, the threshold of genome-wide significance was set at *p* < 5 × 10^−6^. In the mediation analysis, we used a threshold of *p* < 5 × 10^−8^ to select IVs for estimating the causal effects of LST and PA on ICV, *p* < 5 × 10^−6^ for the MR of estimating the causal effect of ICV on childhood IQ. We calculated the F-statistic of each SNP and only the SNPs with F-statistic > 10 were retained given that this method carries the risk of introducing weak IVs. We also used linkage disequilibrium clumping (*r*^*2*^ > 0.001, and < 10 MB) to obtain independent SNPs associated with the exposure, and excluded the SNPs with minor allele frequency of < 0.01. For the second assumption, we investigated each instrument SNP in the PhenoScanner GWAS database (Kamat et al., 2019, Staley et al., 2016) and the NHGRI-EBI GWAS catalog database (https://www.ebi.ac.uk/gwas/docs/file-downloads/) to assess any previous associations (*p* < 5 ×10^−8^), to avoid potential confounders (that is, education, body mass index (BMI)/obesity and common psychiatric disorders). In the analysis of the causal relationship between LST and childhood IQ, two SNPs associated with BMI and three SNPs linked to educational attainment (EA) were excluded, leaving 20 SNPs for further analysis. When examining the relationship between LST and ICV, 15 SNPs associated with BMI, EA, and IQ were excluded. For the investigation of the causal link between PA and ICV, four SNPs associated with BMI, IQ, and LST were removed. For the third assumption, we excluded SNPs strongly associated with the outcome (using the same *p* value threshold as for the exposure variable). Besides, palindromic SNPs were also removed after harmonizing the exposure and outcome data. The remaining SNPs were used to perform MR analysis. The complete lists of IVs used for MR tests can be found in supplemental Table S4, S5, S6.

### 2.4 Mendelian randomization analysis

We employed five MR methods to estimate causal effects while accounting for potential heterogeneity and pleiotropy, including MR random-effect inverse-variance weighted (IVW) (Bowden et al., 2017), MR-Egger (Bowden et al., 2015),weighted median (Bowden et al., 2016), weighted mode (Hartwig et al., 2017), and MR robust adjusted profile score (MR-RAPS) (Zhao et al., 2020). The random-effects IVW method was employed as the main statistical analysis because it offers the most precise estimates. This method combines the ratios of SNP-exposure to SNP-outcome in a random-effects meta-analysis to assess the causal relationship between exposure and outcome. However, this method constrains the regression intercept to zero, relying on the assumption that there is no directional horizontal pleiotropy. Therefore, we employed four other methods to complement and enhance the robustness of the results. The MR-Egger approach addresses pleiotropy by including an intercept term in the model, which enables it to detect and adjust for directional pleiotropy, though this comes at the cost of reduced statistical power (Bowden et al., 2015). The weighted median method can produce valid causal effects as long as at least half of the weight in the analysis is derived from valid IVs (Bowden et al., 2016). The weighted mode method provides consistent estimates when the relaxed IV assumption results in less bias and a lower type I error rate (Hartwig et al., 2017). The MR-RAPS method can provide robust causal estimates by accounting for both systematic and idiosyncratic pleiotropy, particularly in analyses involving numerous weak instruments (Zhao et al., 2020).

#### 2.4.1 Mediating Mendelian Randomization Analysis

To evaluate potential mediation effects, we implemented a two-step MR analysis. First, we selected IVs for LST or PA to estimate their causal effects on the putative mediator (ICV). Second, we used IVs for ICV to assess its causal relationship with childhood IQ. Furthermore, we used the "product of coefficients" method to assess the indirect effect of LST (or PA) on childhood IQ through ICV, deriving the standard error of the indirect effect using the delta method.

### 2.5 Sensitivity analysis

To enhance the reliability of the genetic IVs, we first performed Cochran’s Q test to detect heterogeneity and then used the MR Pleiotropy RESidual Sum and outliers (MR-PRESSO) test (Verbanck et al., 2018) to exclude any outlying SNPs before conducting the final MR analysis. We further performed the MR-Egger intercept test to assess horizontal pleiotropy. Additionally, we performed a leave-one-out analysis to assess whether the observed causal effect was largely driven by any single SNP. We manually checked the genetic IVs of each exposure, and found no overlapped SNPs among exposure phenotypes, avoiding potential interference from overlapping genetic IVs.

### 2.6 Statistics

All analyses were performed using the Two-Sample MR package (version 0.6.6) in R (version 4.4.1). A Bonferroni corrected *p* value threshold was set. The significant estimates should satisfy the following criteria: The *p* value derived from IVW method was < 1.25 × 10^−2^ (0.05/4) in the bidirectional MR analysis, and was < 1.67 × 10^−2^ (0.05/3) in the two-step MR analysis; no heterogeneity was identified by the Cochran’s Q test after removing the outlying SNPs using MR-PRESSO; the *p* values of MR-Egger intercept was greater than 0.05, indicating the absence of horizontal pleiotropy; The leave-one-out plot indicated that the estimate was not influenced by any single SNP. For GWAS summary results of ICV, which provided unstandard beta and SE, we estimated a standard beta and se from the statistic *p* value and allele frequency. In brief, we calculated the z score from statistic *p* value, the calculated standard beta and se using z score and allele frequency.

## 3 Results

### 3.1 Forward Mendelian randomization

**Fig. 2:**
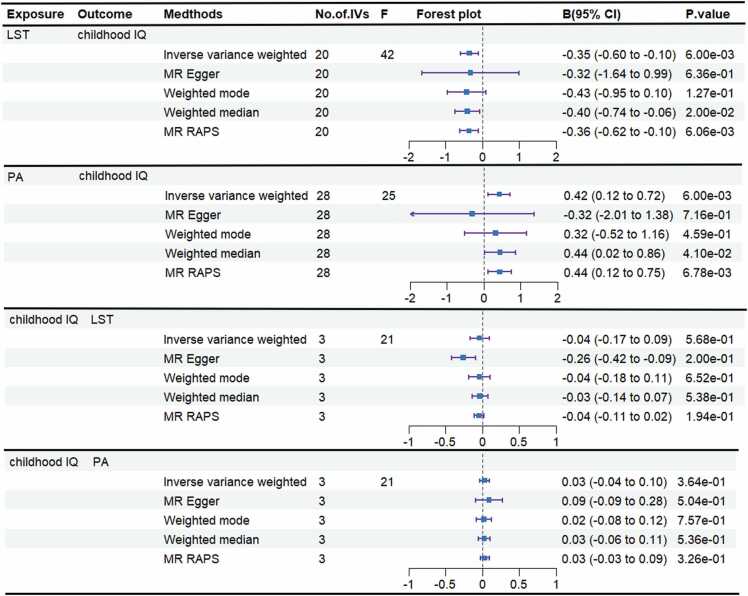
The causal results in the bidirectional two-sample MR analysis. Significant results all met the following criteria: the *p* value derived from IVW method was < 1.25 × 10^−2^ (0.05/4, after Bonferroni correction); no heterogeneity remained after outlier removal using MR-PRESSO (Cochran’s Q test, *p* > 0.05); No evidence of horizontal pleiotropy (MR-Egger intercept and MR-PRESSO global tests *p* > 0.05); the estimate was not biased by any single SNP in leave-one-out analysis. LST = Leisure Screen Time; PA = Moderate-to-Vigorous Physical Activity; IVs = instrumental variables; MR-PRESSO = MR Pleiotropy Residual Sum and Outlier.

In the forward Mendelian randomization (MR) analysis, we used the random-effect inverse-variance weighted (IVW) method and identified that leisure screen time (LST) reduced childhood IQ levels (*β* = −0.35, 95 % CI: [0.60, 0.10], *p* = 5.59 ×10^−3^), while moderate to vigorous physical activity (PA) was beneficial for enhancing childhood IQ (*β* = 0.42, 95 % CI: [0.12, 0.72], *p* = 6.26 × 10^−3^), the other MR methods supported this association, as detailed in Fig. 2 and supplemental Table S7. No heterogeneity was identified among the instrumental variables (IVs) for LST based on the Cochran's Q test (*p*_LST_ = 0.57, *p*_PA_ = 0.49, supplemental Table S7). We used the MR Egger's intercept method to test for horizontal pleiotropy, and found no horizontal pleiotropy (*p*_LST_ = 0.97, *p*_PA_ = 0.39). The scatter plot is shown in supplemental Fig. S1, and the leave-one out analysis confirmed that the estimates were not driven by any single SNP (supplemental Fig. S1). The genetic IVs for the LST and PA can be found in the supplemental Table S8 and Table S9, respectively.

### 3.2 Reverse Mendelian randomization

In the reverse MR analysis, we explored the causal effects of childhood IQ on LST and PA, treating childhood IQ as the exposure and LST and PA as the outcomes. We found no evidence of a causal relationship between childhood IQ either LST (IVW, *β* = −0.04, 95 % CI: [−0.17, 0.09], *p* = 0.57; supplemental Table S10) or PA (IVW, *β* = 0.03, 95 % CI: [-0.04, 0.10], *p* = 0.36; supplemental Table S10). No heterogeneity or horizontal pleiotropy was detected for these exposure-outcome pairs using Cochran’s Q test, MR-Egger intercept test and MR-PRESSO global test (supplemental Table S10). The scatter plots are shown in supplemental Fig. S1, and the estimates were not biased by any SNP in the leave-one-out analysis (supplemental Fig. S1). According to the results, we found no evidence of reverse causality. The genetic IVs for the childhood IQ can be found in the supplemental Table S11.

### 3.3 Two-step mediation Mendelian randomization

Given the LST and PA play critical roles in the prevention and management of brain volume (Spartano et al., 2019), and brain volume has always been considered as one of the important factors affecting childhood IQ (Andreasen et al., 1993). We hypothesized that Brain volume-related measurements might mediate the causal effects of PA and LST on childhood IQ. To test this hypothesis, we conducted a two-step MR analysis to investigate the mediating pathways from LST (or PA) to childhood IQ via intracranial volume (ICV), a commonly used index to measure the head size (Caspi et al., 2020).

In the first step, we used genetic IVs for LST (supplemental Table S12) and PA (supplemental Table S13) to estimate their causal effects on ICV. We identified a significant negative association between LST and ICV, where increased LST was associated with reduced ICV (IVW, *β* = −0.23, 95 % CI: [-0.38, −0.07], *p* = 3.97 ×10^−3^). No significant association was observed for PA. No heterogeneity was detected among the IVs for LST or PA based on the Cochran's Q test (*p*_LST_ = 0.06). We used the MR Egger's intercept method to test for horizontal pleiotropy, and found no horizontal pleiotropy (*p*_LST_ = 0.31). The scatter plot is shown in supplemental Fig. S2, and the estimate was not biased by any SNP in the leave-one out analysis (supplemental Fig. S2).

**Fig. 3:**
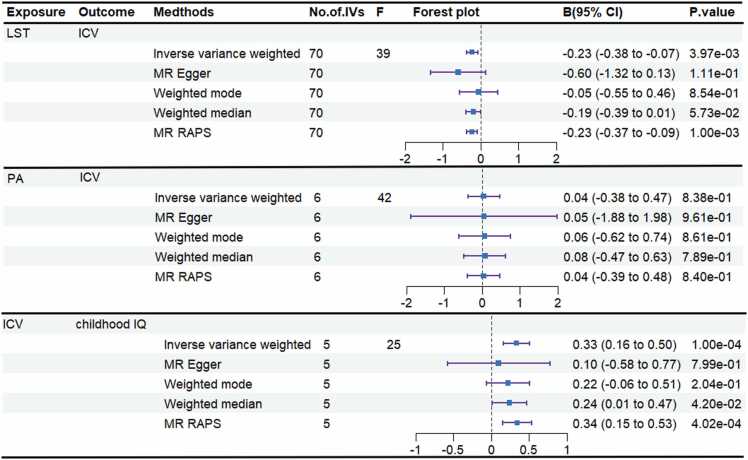
The causal results in the two-step MR analysis. Significant results all met the following criteria: the *p* value derived from IVW method was < 1.67 × 10^−2^ (0.05/3, after Bonferroni correction); no heterogeneity remained after outlier removal using MR-PRESSO (Cochran’s Q test, *p* > 0.05); No evidence of horizontal pleiotropy (MR-Egger intercept and MR-PRESSO global tests *p* > 0.05); the estimate was not biased by any single SNP in leave-one-out analysis. LST = Leisure Screen Time; PA = Moderate-to-Vigorous Physical Activity; IVs = instrumental variables; ICV = Intracranial Volume; MR-PRESSO = MR Pleiotropy Residual Sum and Outlier.

In the second step, we explored the causal effects of ICV on childhood IQ using genetic IVs for ICV (supplemental Table S14). We found a significant positive causal effect of ICV on childhood IQ (IVW, *β* = 0.33, 95 % CI: [0.16, 0.51], *p* = 1.36 × 10^−4^). No heterogeneity (Cochran's Q test, *p* = 0.46) or horizontal pleiotropy (MR-Egger intercept test, *p* = 0.53) was detected. Detailed results can be found in Fig. 3 and Table S15. The scatter plot is shown in supplemental Fig. S2, and the estimate was not biased by any SNP in the leave-one out analysis (supplemental Fig. S2).

Finally, we estimated the mediation effect of LST on childhood IQ via ICV and found a significant mediation effect (*β* = −0.08, 95 % CI: [-0.15, −0.02], *p* = 0.02), accounting for 21.69 % (95 % CI: [15.25 %, 28.13 %]) of the total effect (Table 1).

**Table 1:** The mediation effect of LST on childhood IQ via ICV.

Mediator	Total effect	Direct effect A	Direct effect B	Mediation effect	*p*	mediation proportion (%)
	*β* (95 %IC)	*β* (95 %IC)	*β* (95 %IC)	*β* (95 %IC)		(95 %IC)
ICV	−0.35[−0.60, −0.10]	−0.23[−0.38, −0.07]	0.33[0.16, 0.51]	−0.08[−0.15, −0.02]	0.02	21.69 [15.25, 28.13]

‘Total effect’ indicates the effect of LST on childhood IQ, ‘direct effect A’ indicates the effect of LST on ICV; ‘direct effect B’ indicates the effect of ICV on childhood IQ and ‘mediation effect’ indicates the effect of LST on childhood IQ through ICV; Total effect, direct effect A and direct effect B were derived by IVW; mediation effect was derived by using the delta method. All statistical tests were two-sided. *p* < 0.05 was considered significant.

## 4 Discussion

Using Mendelian randomization (MR), we examined the causal effects of leisure screen time (LST) and moderate to vigorous physical activity (PA) on childhood intelligence (IQ), while investigating intracranial volume (ICV) as a potential mediator. Our results demonstrate that genetically predicted PA confers a protective effect on childhood IQ, whereas LST exerts a detrimental effect. These estimates were consistent across different MR analyses. Mediation analysis further revealed that ICV partially mediated the causal effect of LST on childhood IQ.

The protective effect of PA on cognitive development aligns with a broad literature demonstrating that regular physical activity supports brain health and cognitive performance throughout the lifespan. Meta‑analyses and cohort studies indicate that habitual PA is associated with preserved executive function, memory, and processing speed, and may confer long‑term benefits when initiated in early life or midlife (Desai et al., 2023, Erickson et al., 2022; Hillman et al., 2021; Kumar et al., 2022; Wang et al., 2023). Intervention trials further suggest that structured exercise programs can enhance cognitive outcomes in both healthy and at‑risk populations (Li et al., 2023, Yang et al., 2022).

Conversely, prolonged LST exhibited a negative causal relationship with IQ. This observation is consistent with observational evidence linking excessive screen use to adverse structural and functional brain changes, including reduced grey matter volume and disrupted neural connectivity in regions subserving attention and executive control (Hutton et al., 2020, Paulus et al., 2019). Longitudinal studies further associate early and sustained high screen exposure with poorer attention, executive functioning, and academic performance (Law et al., 2023, Vohr et al., 2021). Although certain digital activities (e.g., educational games) may yield cognitive benefits, overall evidence indicates that excessive passive and multitasking screen behaviors undermine cognitive development (Jia et al., 2024, Melchior et al., 2022; Wang et al., 2024; Yang et al., 2024).

Furthermore, we observed a positive causal effect of ICV on childhood IQ. This result aligns with robust evidence demonstrating that larger brain size, as indexed by ICV, correlates with superior fluid intelligence and executive function throughout the lifespan (MacLullich et al., 2002, Manfro et al., 2021, Roussotte et al., 2012, van Loenhoud et al., 2022). Shared genetic influences on ICV and cognitive ability further support brain volume as a key determinant of intellectual capacity (Adams et al., 2016, Hagenaars et al., 2016, Haworth et al., 2019).

Our two-step MR mediation analysis reveals that ICV partially mediates the negative effect of LST on childhood IQ. Specifically, we observe a negative causal effect of LST on ICV alongside a positive causal influence of ICV on childhood IQ, consistent with a mechanistic pathway whereby excessive screen exposure impedes brain growth and, in turn, diminishes cognitive capacity. While prior neuroimaging research has documented associations between excessive screen exposure and reduced brain volume, particularly in temporal‑parietal and frontal regions involved in attention and higher‑order cognition (Ali et al., 2024, Giedd, 2020, Hutton et al., 2022, Mewton et al., 2023), our study is the first to leverage genetic instruments to establish ICV as a causal mediator in this relationship. Such structural alterations likely contribute to the negative causal effect of LST on ICV revealed in our analysis.

Conversely, physical activity has been linked to increased brain volume in regions supporting memory and executive function, such as the hippocampus and prefrontal cortex across diverse age groups (Erickson et al., 2014, Firth et al., 2018, Gu et al., 2020; Hillman et al., 2021; Raji et al., 2024; Sewell et al., 2024; Spartano et al., 2019; Tarumi et al., 2022; Valkenborghs et al., 2019; Voss et al., 2013; Wood et al., 2016). These volumetric enhancements may underlie the protective effect of PA on IQ.

To the best of our knowledge, our study is the first to propose that ICV may mediate the causal relationship between LST and IQ. Specifically, we suggest that prolonged engagement in screen-based leisure activities could influence brain structure, particularly ICV, which in turn may affect cognitive abilities, including IQ. This finding highlights potential neurological mechanisms through which screen time impacts cognitive function, offering novel insights into how lifestyle factors shape brain development and intelligence. However, this study has several limitations. First, we cannot completely rule out violations of the assumptions of independence and exclusion restrictions, especially regarding pleiotropy. Despite this, we used several methods to infer robust causal estimates, including MR-PRESSO, weighted median, weighted mode, MR-Egger, and leave-one-out sensitivity analyses. At the same time, the causal effects are estimated through genetic variants rather than randomized controlled trials, so the results need to be interpreted with caution. Second, the results of MR analysis may be affected by sample selection bias. We conducted our analysis using two-sample MR, with LST and PA GWAS data including the UK Biobank database, which has an older sample age, while the sample for IQ is younger, thus introducing age bias. Moreover, because both exposure and outcome GWAS were conducted predominantly in individuals of European ancestry, the extent to which our findings generalize to ancestrally diverse populations (for example, African, Asian, or admixed groups) remains unclear. Lastly, due to environmental and social factors, such as cultural differences, genetic nurture, and population structure, MR estimates for unrelated individuals may be biased. This bias could be avoided by using within-family GWAS in future studies.

Our study utilized large-scale exposure and outcome GWAS datasets to conduct MR analysis to investigate the causal effects of LST and PA on childhood IQ. We found robust genetic evidence for a protective association between PA and childhood IQ, while a risk association between LST and childhood IQ, and to some extent, ICV mediated the causal effect of LST on childhood IQ. These findings highlight the critical need to manage and regulate children's media usage while also promoting increased PA. Taking proactive steps to support the healthy development of children in the digital age is essential for their well-being and growth.

## CRediT authorship contribution statement

**Feng Junjiao:** Writing – review & editing, Writing – original draft, Visualization, Validation, Supervision, Methodology, Investigation, Funding acquisition, Formal analysis, Data curation, Conceptualization. **Wan Yi:** Writing – original draft, Visualization, Validation, Methodology, Formal analysis, Data curation. **Liang Zhang:** Writing – review & editing.

## Ethical approval and consent to participate

This MR study utilized de-identified summary-level data that are publicly accessible. Informed consent and ethical approval were obtained for all original GWAS studies, and therefore, no further ethical approval was necessary for this study. All authors approve the publication of the manuscript in its current form.

## Declaration of Competing Interest

All authors declare no conflicts of interest.

## Data Availability

I have shared the link to my data/code at the Attach Flie step.
