# Supplementary material for: Causal effects of physical activity and screen time on childhood intelligence via Mendelian randomization: The mediating role of intracranial volume
Source: Dev Cogn Neurosci. 2025 Jun 20;74:101586. doi: 10.1016/j.dcn.2025.101586 (PMC12221466; doi:10.1016/j.dcn.2025.101586)

**Supplemental Figures**

# **Fig. S1** Scatter plots and leave-one-out plots of significant estimates in both forward and reverse Mendelian randomization analyses. LST, PA, childhood IQ.

A B


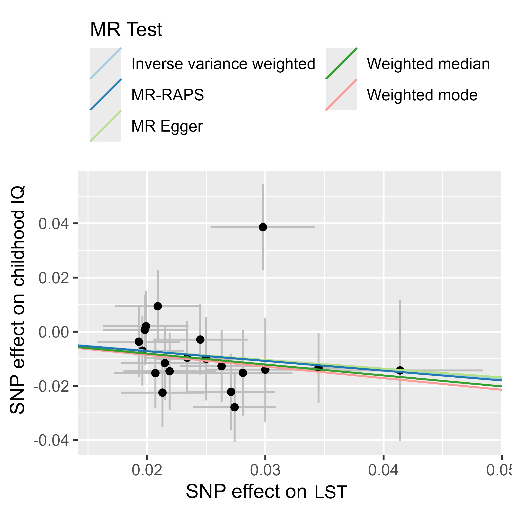

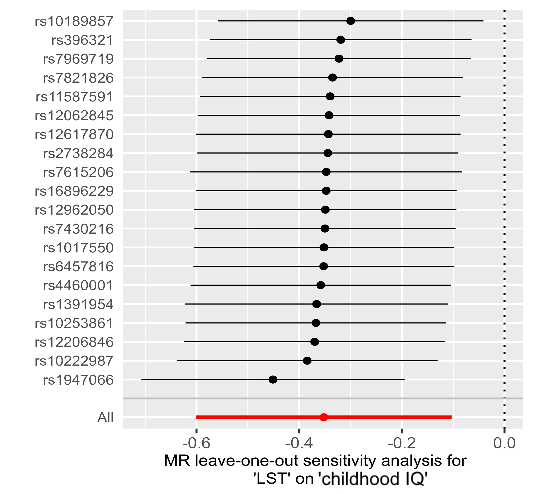


C D


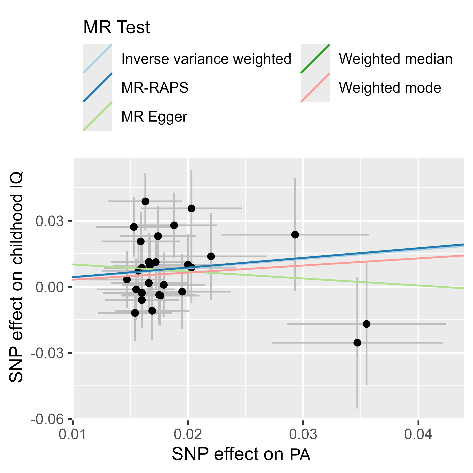

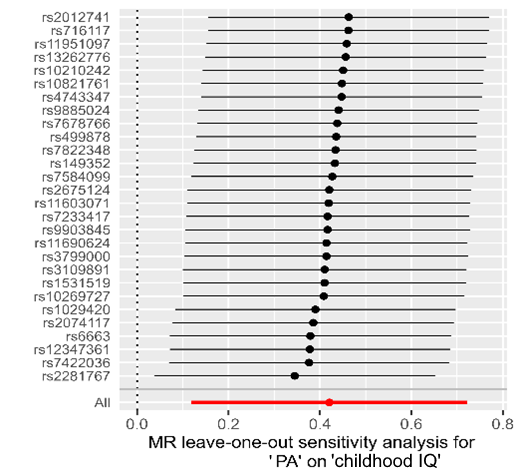


E F


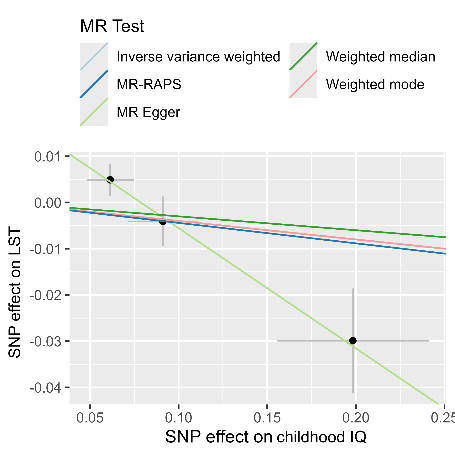

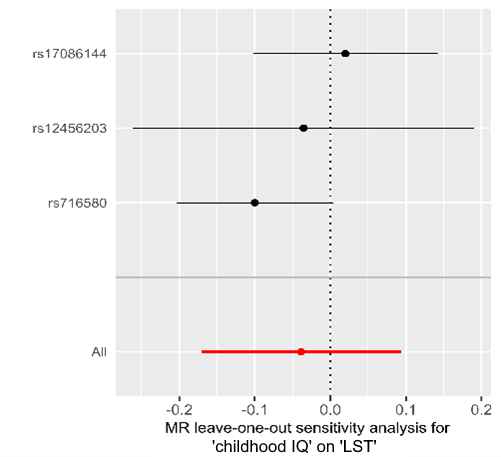


G H


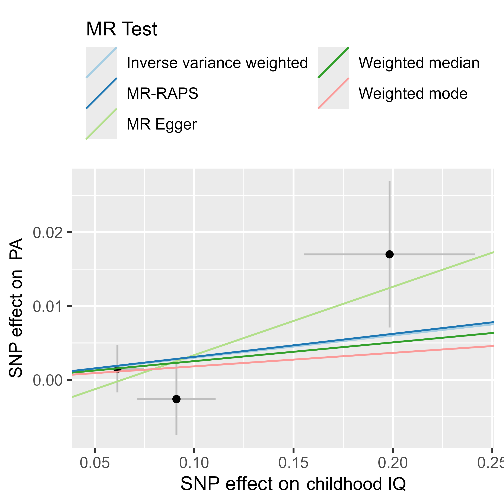

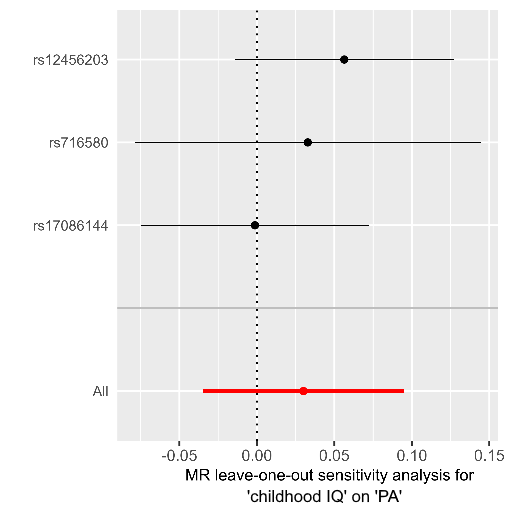


# **Fig. S2** Scatter plots and leave-one-out plots of significant estimates in two-step Mendelian randomization analyses. LST, MVPA, ICV, childhood IQ.

A B


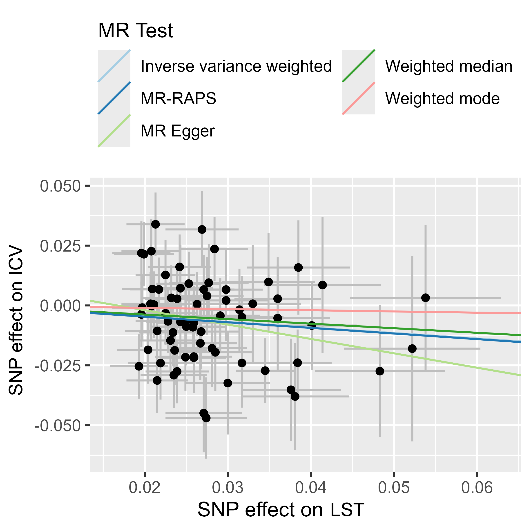

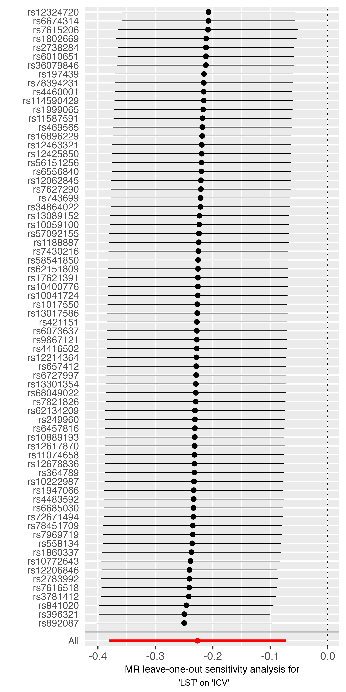


C D


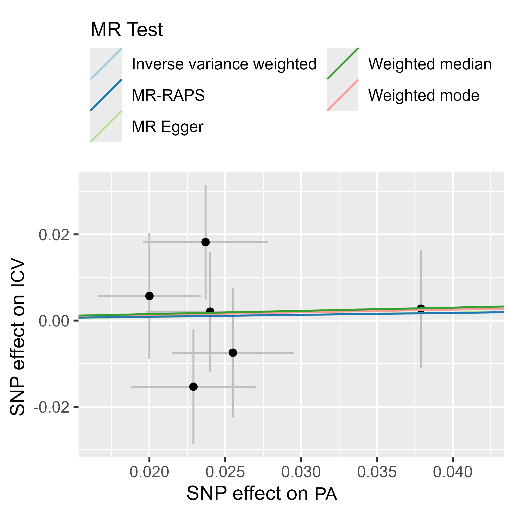

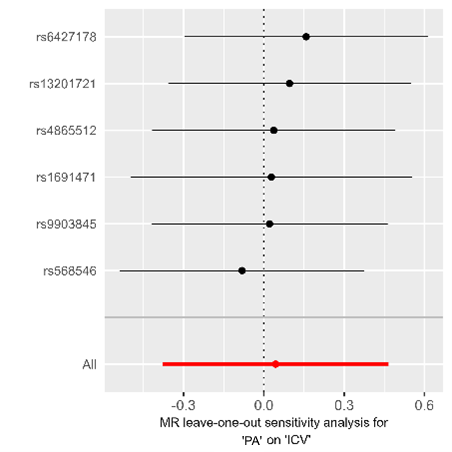


E F


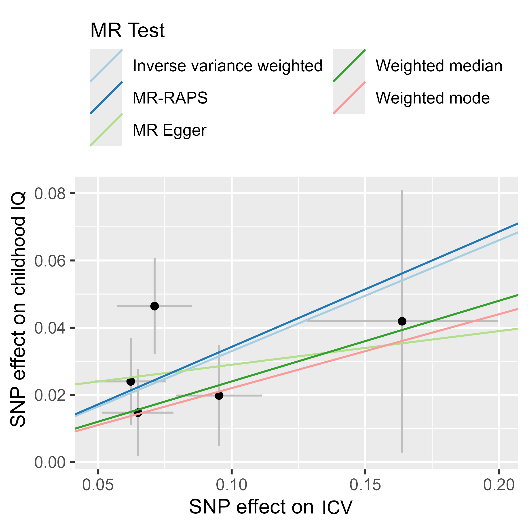

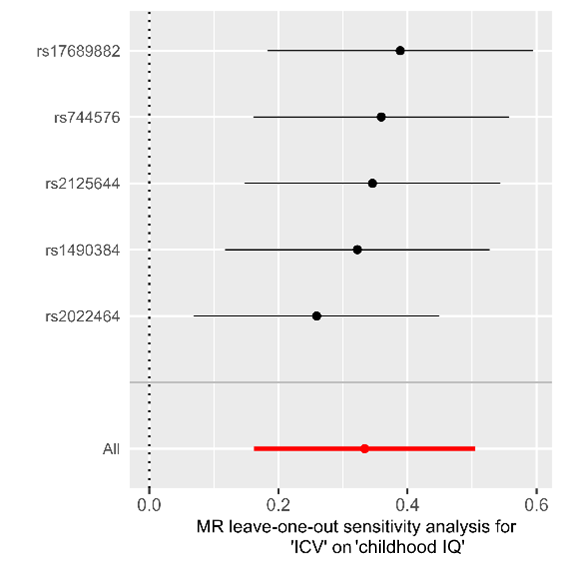

Supplement: Supplementary file 1 — Supplementary material [file mmc1.docx]
