# Supplementary material for: Causal effects of physical activity and screen time on childhood intelligence via Mendelian randomization: The mediating role of intracranial volume
Source: Dev Cogn Neurosci. 2025 Jun 20;74:101586. doi: 10.1016/j.dcn.2025.101586 (PMC12221466; doi:10.1016/j.dcn.2025.101586)
Supplement: Supplementary file 2 — Supplementary material [file mmc2.doc]

| **Supplementary tables** | |
| --- | --- |
| **Table** | **Contents** |
| Table S1 | Descriptions of study cohorts participating in the genome-wide association studies meta-analysis to identify genetic variants with PA and LST. |
| Table S2 | Description of study cohorts participating in meta-analyses of genome-wide association studies to identify genetic variants associated with childhood IQ. |
| Table S3 | Description of study cohorts participating in meta-analyses of genome-wide association studies to identify genetic variants associated with ICV. |
| Table S4 | Information of instrumental variables for all exposure-outcome pairs in forward Mendelian randomization analyses. |
| Table S5 | Information of instrumental variables for all exposure-outcome pairs in reverse Mendelian randomization analyses. |
| Table S6 | Information of instrumental variables for all exposure-outcome pairs in two-step Mendelian randomization analyses. |
| Table S7 | Forward Mendelian randomization analysis results. |
| Table S8 | Summary information on LST SNPs used as genetic instruments for the Mendelian randomization analyses with childhood IQ. |
| Table S9 | Summary information on PA SNPs used as genetic instruments for the Mendelian randomization analyses with childhood IQ. |
| Table S10 | Reverse Mendelian randomization analysis results. |
| Table S11 | Summary information on childhood IQ SNPs used as genetic instruments for the Mendelian randomization analyses with PA and LST. |
| Table S12 | Summary information on LST SNPs used as genetic instruments for the Mendelian randomization analyses with ICV. |
| Table S13 | Summary information on PA SNPs used as genetic instruments for the Mendelian randomization analyses with ICV. |
| Table S14 | Summary information on ICV SNPs used as genetic instruments for the Mendelian randomization analyses with childhood IQ. |
| Table S15 | Two-step Mendelian randomization analysis results. |

| **Table S1. Descriptions of study cohorts participating in the genome-wide association studies meta-analysis to identify genetic variants with PA, LST** | | | | | | |
| --- | --- | --- | --- | --- | --- | --- |
| **Cohort** | **Ancestry** | **Sex** | **Total N** | **Leisure screen time (hours/day)** | **PA** | **Mean Age (SD)** |
|  |  |  |  | **Mean (SD)** | **Active' vs. 'inactive'** |  |
| ALSPAC-mothers | European | men | . | . | . | . |
|  |  | women | 4,545 | 2.59(1.58) | 1231/3290 | 36.43(4.48) |
| ALSPAC-offspring | European | men | 1,175 | 1.81(1.01) | . | 16.70(0.25) |
|  |  | women | 1,618 | 1.67(0.96) | . | 16.67(0.23) |
| ARIC | European | men | 3,902 | . | 3327/1490 | 53.91(5.46) |
|  |  | women | 3,752 | . | 3699/595 | 52.86(5.36) |
| B58C-WTCCC2 | European | men | 1,394 | 2.20(1.10) | 1322/34 | 45.10(0.38) |
|  |  | women | 1,307 | 2.20(1.20) | 1219/52 | 45.10(0.40) |
| B58C- T1DGC | European | men | 1,235 | 2.20(1.10) | 1181/19 | 45.30(0.30) |
|  |  | women | 1,294 | 2.20(1.10) | 1198/54 | 45.30(0.30) |
| B-PROOF | European | men | 1,266 | . | 692/574 | 73.40(6.10) |
|  |  | women | 1,257 | . | 71/486 | 74.50(6.90) |
| CARDIA | European | men | 758 | 1.82(1.59) | 500/258 | 30.62(3.31) |
|  |  | women | 853 | 1.60(1.45) | 529/324 | 30.55(3.36) |
| CHS | European | men | 1,277 | . | 945/332 | 72.98(5.64) |
|  |  | women | 1,989 | . | 1329/660 | 71.92(5.16) |
| CoLAUS round 1 | European | men | 2,542 | . | 1641/901 | 52.98(10.76) |
|  |  | women | 2,862 | . | 1867/996 | 53.93(10.72) |
| CoLAUS round 2 | European | men | 1,648 | 1.14(1.15) |  | 57.93(10.59) |
|  |  | women | 1,949 | 0.93(1.11) |  | 58.73(10.42) |
| DNBC | European | case women | 830 | . | 284 / 546 | 29.72(4.44) |
|  |  | control women | 969 | . | 330/ 639 | 30.31(3.99) |
| EGCUT | European | Metabo men | 816 | . | 469/337 | 58.85(10.93) |
|  |  | Metabo women | 1,248 | . | 695/534 | 58.12(12.00) |
|  |  | 370cnv men | 1,057 | . | 812/146 | 38.12(15.40) |
|  |  | 370cnv women | 1,130 | . | 891/154 | 39.85(15.28) |
|  |  | omni men | 3,032 | 1.84(1.34) | 1894/1100 | 49.64(9.53) |
|  |  | omni women | 3,834 | 1.84(1.29) | 2339/1457 | 50.19(20.43) |
| EPIC-Norfolk | European | men | 9,793 | 3.08(1.45) | 3499 / 2660 | 59.59(9.24) |
|  |  | women | 11,251 | 3.14(1.43) | 4200 / 3263 | 58.77(9.29) |
| FAMHS | European | men | 1,744 | 2.19(1.54) | 1093/651 | 51.00(13.87) |
|  |  | women | 1,929 | 2.10(1.48) | 1207/720 | 52.41(13.44) |
| Fenland-OMICS | European | men | 4,248 | 2.67(1.43) | 2590/1073 | 48.81(7.41) |
|  |  | women | 4,746 | 2.68(1.46) | 2426/1213 | 48.90(7.33) |
| FRAM | European | men | 3,312 | . | 3208/104 | 45.37(10.93) |
|  |  | women | 3,713 | . | 3652/61 | 44.99(10.75) |
| FUSION | European | case men | 612 | . | 67/542 | 61.97(7.30) |
|  |  | case women | 463 | . | 24/435 | 63.57(7.74) |
|  |  | control men | 454 | . | 111/337 | 61.71(7.61) |
|  |  | control women | 453 | . | 72/377 | 61.94(7.18) |
| GENOA | European | men | 491 | 2.41(1.52) | . | 59.48(10.05) |
|  |  | women | 629 | 2.41(1.52) | . | 58.64(10.20) |
| GOOD | European | men | 938 | 3.18(2.18) | 602/339 | 18.90(0.56) |
| GOYA | European | case men | 625 | . | . | 43.00(6.20) |
|  |  | control men | 715 | . | . | 47.70(8.50) |
| GRAPHIC | European | men | 508 | 5.73(1.46) | 231 / 205 | 53.81(4.24) |
|  |  | women | 509 | 5.71(1.55) | 218 / 211 | 51.90(4.35) |
| HEALTH2006 | European | men | 1,415 | . | 723/692 | 50.33(12.78) |
|  |  | women | 1,771 | . | 1063/708 | 48.82(13.03) |
| HPFSaffy | European | case men | 1,522 | 1.60(1.30) | 741/814 | 55.90(8.60) |
|  |  | control men | 2,050 | 1.50(1.20) | 846/1204 | 55.90(8.50) |
| HPFSillumina | European | case men | 644 | 1.40(1.20) | 383/267 | 53.50(8.90) |
|  |  | control men | 726 | 1.50(1.20) | 453/273 | 54.80(8.50) |
| HPFSomni | European | case men | 924 | 1.50(1.20) | 545/379 | 54.70(8.80) |
|  |  | control men | 898 | 1.40(1.20) | 556/342 | 54.50(8.80) |
| HRS | European | men | 1,524 | 3.08(1.84) | . | 67.66(10.12) |
|  |  | women | 2,135 | 2.99(1.87) | . | 66.97(11.16) |
| inCHIANTI | European | men | 537 | . | . | 67.03(15.38) |
|  |  | women | 666 | . | . | 68.98(15.59) |
| KORA S3 | European | men | 813 | . | 385/428 | 52.96(10.09) |
|  |  | women | 831 | . | 324/507 | 52.09(10.08) |
| KORA S4 | European | men | 877 | . | 380/497 | 54.19(8.92) |
|  |  | women | 928 | . | 440/488 | 53.64(8.80) |
| KORCULA | European | men | 313 | . | . | 57.10(14.07) |
|  |  | women | 557 | . | . | 55.13(13.46) |
| LifeLines | European | men | 4,825 | . | 1090/3735 | 48.43(11.22) |
|  |  | women | 6,980 | . | 1099/5881 | 48.18(10.86) |
| MDC-CVA | European | men | 2,041 | . | 1525/516 | 57.47(5.96) |
|  |  | women | 2,955 | . | 2160/795 | 57.48(5.93) |
| MESA | European | men | 1,285 | 1.91(1.37) | 863/422 | 62.83(10.08) |
|  |  | women | 1,400 | 2.02(1.52) | 821/578 | 62.67(10.23) |
| METSIM | European | men | 8,967 | . | 4237 / 4730 | 57.49(7.12) |
| NESDA | European | men | 638 | 3.00(1.90) | . | 44.30(12.30) |
|  |  | women | 1,237 | 3.00(1.80) | . | 41.50(12.90) |
| NFBC1966 | European | men | 2,123 | . | 1445/672 | 31.00(0.00) |
|  |  | women | 2,234 | . | 1705/527 | 31.00(0.00) |
| NHSaffy | European | case women | 1,849 | 2.10(1.90) | 866/989 | 54.50(6.80) |
|  |  | control women | 2,499 | 1.90(1.60) | 1306/1193 | 54.50(6.80) |
| NHSillumina | European | case women | 1,719 | 1.90(1.70) | 898/821 | 54.50(6.70) |
|  |  | control women | 1,721 | 1.90(1.60) | 899/822 | 54.60(6.50) |
| NHSomni | European | case women | 1,194 | 1.90(1.80) | 619/575 | 54.40(6.70) |
|  |  | control women | 2,075 | 1.90(1.60) | 1002/1073 | 54.70(6.60) |
| NSPHS | European | men | 478 | . | 261/212 | 50.96(19.97) |
|  |  | women | 550 | . | 285/259 | 49.97(20.25) |
| ORCADES | European | men | 525 | . | . | 49.08(13.10) |
|  |  | women | 737 | . | . | 47.92(13.14) |
| QIMR | European | men | 896 | . | 849 / 548 | 35.01(11.87) |
|  |  | women | 1,998 | . | 1356 / 1271 | 38.86(14.09) |
| RSⅠ | European | men | 1,457 | . | 386/1071 | 71.73(6.47) |
|  |  | women | 2,209 | . | 590/1619 | 72.72(7.27) |
| RSⅡ | European | men | 904 | . | 313/591 | 63.98(7.64) |
|  |  | women | 1,096 | . | 410/686 | 64.50(8.13) |
| SHIP | European | men | 1,992 | . | . | 50.80(16.40) |
|  |  | women | 2,056 | . | . | 48.60(16.00) |
| SHIP-TREND | European | men | 431 | . | 224 / 207 | 50.10(14.24) |
|  |  | women | 553 | . | 281 / 272 | 50.15(13.26) |
| Twingen | European | men | 2,317 | . | . | 32.19(0.00) |
|  |  | women | 2,727 | . | . | 31.30(0.00) |
| TwinsUK | European | women | 882 | . | . | 61.11(9.68) |
| UK BioBank | European | men | 205,133 | 3.64(1.87) | 92703/112430 | 56.98(8.11) |
|  |  | women | 243,769 | 4.02(2.01) | 109418/134351 | 56.58(7.95) |
| VIS | European | men | 383 | . | . | 55.87(14.92) |
|  |  | women | 522 | . | . | 56.42(15.91) |
| WGHS | European | women | 22,865 | 1.84(2.02) | 10238/12627 | 54.20(7.10) |
| WHI | European | (GECCO+HIPFX)women | 2,798 | . | 1,153/1,645 | 64.26(6.61) |
| YFS | European | men | 1,180 | 2.00(1.17) | 959 / 218 | 32.74(5.70) |
|  |  | women | 1,396 | 1.76(1.09) | 1194 / 195 | 32.65(5.68) |
|  |  | SUM | 661,399 |  |  |  |
| This table is modified from Zhe Wang, Andrew Emmerich, Nicolas J. Pillon, et al. Genome-wide association analyses of physical activity and sedentary behavior provide insights into underlying mechanisms and roles in disease prevention.Nature Genetics volume 54, pages1332–1344 (2022) | | | | | | |
| Note: "." indicates that detailed information was not found. | | | | | | |

| **Table S2. Descriptions of study cohorts participating in the genome-wide association studies meta-analysis to identify genetic variants with childhood IQ** | | | | |
| --- | --- | --- | --- | --- |
| **Cohort** | **Ancestry** | **Total N** | **Intelligence measure** | **Mean Age** |
| Avon Longitudinal Study of Parents and Children (ALSPAC) | UK | 15247 | WISC-III | 9 |
| Lothian Birth Cohort 1921 (LBC1921) | UK | 464 | Moray House Test No. 12 | 11 |
| Lothian Birth Cohort 1936 (LBC1936) | UK | 947 | Moray House Test No. 12 | 11 |
| Brisbane Adolescent Twins Study, Queensland Institute of Medical Research (QIMR) cohort | Australia | 1752 | MAB Full-scale IQ | 16 |
| Western Australian Pregnancy Cohort (Raine) Study | Australia | 936 | g score | 10 |
| Twins of Early Development Study (TEDS) | UK | 2,825 | g score | 12 |
| Generation Rotterdam Study (GenR) | The Netherlands | 1442 | SON-R 2,5–7 | 6 |
| Netherlands Twin Register (NTR) | The Netherlands | 739 | RAKIT, WISC-R, WISC-R-III, WAIS-III | 13 |
| University of Minnesota Study (UMN) | USA | 3367 | WISC-R, WAIS-R | 14 |
| This table is modified fromB Benyamin1,2,26, BSt Pourcain3,26, OS Davis4,26, G Davies5,26, NK Hansell2, et al. Childhood intelligence is heritable, highly polygenic and associated with FNBP1L.Mol Psychiatry. 2014 February; 19(2): 253–258. | | | | |

| **Table S3. Descriptions of study cohorts participating in the genome-wide association studies meta-analysis to identify genetic variants associated with ICV** | | | | |
| --- | --- | --- | --- | --- |
| **Cohort** | **Study Design** | **Ancestry** | **Total N** | **Mean Age (SD)** |
| **AddNeuroMed** | Case-control (AD, MCI and health controls) | European | 357 | 74.4 (6.4) |
| **ADNI** | Case-Control (AD, MCI, healthy control) | European | 747 | 75.4 (6.9) |
| **ADNI2GO** | Case-Control (AD, MCI, healthy control) | European | 362 | 72.8 (7.4) |
| **Betula** | Population-based | European | 353 | 62.3 (13.3) |
| **BFS** | Population-based | European | 220 | 24 (7.9) |
| **BIG** | Population-based | European | 1300 | 22.9 (3.8) |
| **BrainSCALE** | Population-based Twin Study | European | 277 | 10.0 (1.3) |
| **BRCDECC** | Case-control (MDD and healthy controls) | European | 169 | 49.9 (8.6) |
| **EPIGEN** | Epilepsy cases | European | 233 | 38.5 (12.7) |
| **GIG** | Population-based | European | 299 | 24.2 (2.4) |
| **GSP** | Population-based | European | 442 | 21.4 (3.2) |
| **HUBIN** | Case-control (SCZ and healthy controls) | European | 200 | 41.8 (8.1) |
| **IMAGEN** | Population-based | European | 1765 | 14.6 (0.4) |
| **MCIC** | Case-control (SCZ and healthy controls) | European | 170 | 34.0 (11.2) |
| **MooDS** | Population-based | European | 311 | 33.4 (9.8) |
| **MPIP** | Case-control (MDD and healthy controls) | European | 550 | 48.3 (13.3) |
| **NCNG** | Population-based | European | 327 | 51.8 (16.7) |
| **NESDA** | Case-control (Depression, Anxiety, and healthy controls) | European | 231 | 37.8 (10.1) |
| **neuroIMAGE** | ADHD cases | European | 154 | 17.0 (2.5) |
| **NTR - Adults** | Population-based Twin Study | European | 400 | 29.7 (10.7) |
| **OATS** | Population-based Twin study | European | 364 | 70.5 (5.1) |
| **PAFIP** | Case-control (SCZ and healthy controls) | European | 117 | 28.4 (8.1) |
| **QTIM** | Population-based Twin Study | European | 845 | 22.5 (3.2) |
| **SHIP** | Population-based | European | 966 | 56.4 (12.6) |
| **SHIP-TREND** | Population-based | European | 858 | 50.0 (13.5) |
| **Sydney MAS** | Population-based | European | 543 | 78.4 (4.7) |
| **TOP** | Case-control (SCZ, BD, other psychoses, and healthy controls) | European | 849 | 34.0 (10.4) |
| **UMCU** | Case-control (SCZ and healthy controls) | European | 279 | 31.9 (11.7) |
| This table is modified from Hibar, D. P., Stein, J. L., Renteria, M. E., Arias-Vasquez, A., Desrivieres, S., Jahanshad, N., et al. Common genetics variants influence human subcortical brain structures. Nature, 520(7546), 224–229. | | | | |

| **Table S4. Information of instrumental variables for all exposure-outcome pairs in forward Mendelian randomization analyses.** | | | |
| --- | --- | --- | --- |
| **Exposure** | **Outcome** | **No. of instrumental variables** | **Instrumental variables** |
| LST | childhood IQ | 20 | rs1017550, rs10189857, rs10222987, rs10253861, rs11587591, rs12062845, rs12206846, rs12617870, rs12962050, rs1391954, rs16896229, rs1947066, rs2738284, rs396321, rs4460001, rs6457816, rs7430216, rs7615206, rs7821826, rs7969719 |
| PA | childhood IQ | 28 | rs10210242, rs10269727, rs1029420, rs10821761, rs11603071, rs11690624, rs11951097, rs12347361, rs13262776, rs149352, rs1531519, rs2012741, rs2074117, rs2281767, rs2675124, rs3109891, rs3799000, rs4743347, rs499878, rs6663, rs716117, rs7233417, rs7422036, rs7584099, rs7678766, rs7822348, rs9885024, rs9903845 |

| **Table S5. Information of instrumental variables for all exposure-outcome pairs in reverse Mendelian randomization analyses.** | | | |
| --- | --- | --- | --- |
| **Exposure** | **Outcome** | **No. of instrumental variables** | **Instrumental variables** |
| childhood IQ | LST | 3 | rs12456203, rs17086144, rs716580 |
| childhood IQ | PA | 3 | rs12456203, rs17086144, rs716580 |
| Due to the number of SNPs in ICV being less than three, even relaxed the threshold(5e-6) we did not present the reverse MR results for ICV. | | | |

| **Table S6. Information of instrumental variables for all exposure-outcome pairs in two-step Mendelian randomization analyses.** | | | |
| --- | --- | --- | --- |
| **Exposure** | **Outcome** | **No. of instrumental variables** | **Instrumental variables** |
| LST | ICV | 70 | rs6674314,rs12324720,rs396321,rs2738284,rs6010651,rs36079846,rs7615206,rs841020,rs4460001,rs892087,rs1802669,rs78394231,rs3781412,rs114590429,rs7616518,rs197439,rs12206846,rs11587591,rs1999065,rs16896229,rs469565,rs6556840,rs12425850,rs12463321,rs56151256,rs2783992,rs12062845,rs7627290,rs743699,rs34864022,rs558134,rs10772643,rs13089152,rs10059100,rs57092155,rs1860337,rs1188887,rs78451709,rs7430216,rs6685030,rs62151809,rs10222987,rs72671494,rs10400776,rs58541850,rs7969719,rs17621391,rs13017586,rs10041724,rs10889193,rs1947066,rs6457816,rs9867121,rs1017550,rs4416502,rs6073637,rs364789,rs421151,rs12678836,rs11074658,rs4483592,rs249960,rs62134209,rs68049022,rs12214364,rs7821826,rs12617870,rs657412,rs13301354,rs6727997 |
| PA | ICV | 6 | rs13201721, rs1691471, rs4865512, rs568546, rs6427178, rs9903845 |
| ICV | childhood IQ | 5 | rs1490384, rs17689882, rs2125644, rs744576, rs2022464 |

| **Table S7. Forward Mendelian randomization analysis results.** | | | | | | | | | | | | | | | | | | | | | | | | |
| --- | --- | --- | --- | --- | --- | --- | --- | --- | --- | --- | --- | --- | --- | --- | --- | --- | --- | --- | --- | --- | --- | --- | --- | --- |
| Note: Causal effects estimated using 5 two-sample Mendelian randomization methods (inverse-variance weighted, MR-Egger, weighted median, weighted mode and MR-RAPS). All statistical tests were two-sided. *P-*value < 1.25E-2 after Bonferroni correction was considered significant. | | | | | | | | | | | | | | | | | | | | | | | | |
| **Exposure** | **Outcome** | ***P*-value of MR-PRESSO global test** | **Cochran’s Q derived *P-*value** | ***P*-value of MR-Egger intercept** | **Inverse-variance weighted** | | | | **MR-Egger** | | | | **Weighted median** | | | | **weighted mode** | | | | **MR-RAPS** | | | |
|  |  |  |  |  | **Estimate (β)** | **95% lower CI** | **95% upper CI** | ***P-*value** | **Estimate (β)** | **95% lower CI** | **95% upper CI** | ***P-*value** | **Estimate (β)** | **95% lower CI** | **95% upper CI** | ***P-*value** | **Estimate (β)** | **95% lower CI** | **95% upper CI** | ***P-*value** | **Estimate (β)** | **95% lower CI** | **95% upper CI** | ***P-*value** |
| LST | childhood IQ | 0.504 | 0.570 | 0.965 | -0.352 | -0.601 | -0.103 | 0.006 | -0.323 | -1.635 | 0.990 | 0.636 | -0.404 | -0.743 | -0.065 | 0.020 | -0.429 | -0.951 | 0.093 | 0.127 | -0.360 | -0.616 | -0.103 | 0.006 |
| PA | childhood IQ | 0.480 | 0.494 | 0.393 | 0.421 | 0.119 | 0.722 | 0.006 | -0.319 | -2.014 | 1.377 | 0.716 | 0.438 | 0.018 | 0.858 | 0.041 | 0.322 | -0.518 | 1.163 | 0.459 | 0.437 | 0.121 | 0.754 | 0.007 |

| **Table S8. Summary information on LST SNPs used as genetic instruments for the Mendelian randomization analyses with childhood IQ** | | | | | | | | | | | | | | | | | | |
| --- | --- | --- | --- | --- | --- | --- | --- | --- | --- | --- | --- | --- | --- | --- | --- | --- | --- | --- |
| **SNP** | **effect_allele.exposure** | **other_allele.exposure** | **effect_allele.outcome** | **other_allele.outcome** | **beta. exposure** | **beta. outcome** | **eaf. exposure** | **eaf. outcome** | **se. outcome** | **pval. outcome** | **se. exposure** | **pval. exposure** | **F-**  **statistics** | **SampleSize** | **Chromosome** | **base pair location(GRCh37/hg19)** | **Gene** | **Functional  anotation** |
| rs1017550 | A | G | A | G | 0.020 | -0.007 | 0.586 | 0.576 | 0.013 | 0.586 | 0.004 | 2.85E-08 | 31.360 | 525491 | 10 | 63587683 | LINC02625 | Intron Variant |
| rs10189857 | A | G | A | G | -0.027 | 0.028 | 0.565 | 0.567 | 0.013 | 0.030 | 0.004 | 7.80E-15 | 61.287 | 525491 | 2 | 60713235 | BCL11A | Intron Variant |
| rs10222987 | A | G | A | G | -0.021 | -0.009 | 0.636 | 0.649 | 0.014 | 0.485 | 0.004 | 9.02E-09 | 33.704 | 523875 | 4 | 185946130 | none | none |
| rs10253861 | A | G | A | G | -0.020 | -0.001 | 0.551 | 0.533 | 0.013 | 0.956 | 0.004 | 1.48E-08 | 32.003 | 525489 | 7 | 8110475 | GLCCI1 | Intron Variant |
| rs11587591 | A | G | A | G | 0.022 | -0.015 | 0.734 | 0.767 | 0.014 | 0.307 | 0.004 | 2.23E-08 | 31.533 | 526365 | 1 | 209762875 | CAMK1G | Intron Variant |
| rs12062845 | A | C | A | C | 0.028 | -0.015 | 0.216 | 0.136 | 0.016 | 0.338 | 0.004 | 2.41E-11 | 44.762 | 526370 | 1 | 98342685 | DPYD | Intron Variant |
| rs12206846 | A | G | A | G | 0.020 | 0.002 | 0.405 | 0.406 | 0.013 | 0.877 | 0.004 | 2.10E-08 | 30.556 | 525490 | 6 | 108238917 | SEC63 | Intron Variant |
| rs12617870 | T | G | T | G | 0.026 | -0.013 | 0.538 | 0.567 | 0.013 | 0.335 | 0.004 | 6.62E-14 | 56.464 | 525491 | 2 | 193746283 | SLC44A3P1 | Intron Variant |
| rs12962050 | A | G | A | G | -0.023 | 0.010 | 0.647 | 0.613 | 0.014 | 0.475 | 0.004 | 1.18E-10 | 42.250 | 525490 | 18 | 35179808 | CELF4 | Intron Variant |
| rs1391954 | T | G | T | G | 0.025 | -0.003 | 0.444 | 0.350 | 0.013 | 0.824 | 0.004 | 1.51E-09 | 37.516 | 442658 | 11 | 88575965 | GRM5 | Intron Variant |
| rs16896229 | T | C | T | C | 0.030 | -0.014 | 0.136 | 0.119 | 0.019 | 0.465 | 0.005 | 3.80E-09 | 34.602 | 523040 | 4 | 18002583 | LCORL | Intron Variant |
| rs1947066 | A | G | A | G | 0.030 | 0.039 | 0.803 | 0.814 | 0.016 | 0.015 | 0.004 | 8.54E-12 | 45.870 | 525491 | 5 | 161101615 | GABRA6 | Intron Variant |
| rs2738284 | A | G | A | G | -0.022 | 0.012 | 0.329 | 0.379 | 0.014 | 0.395 | 0.004 | 7.05E-09 | 33.766 | 523619 | 2 | 217311609 | SMARCAL1 | Intron Variant |
| rs396321 | T | C | T | C | -0.021 | 0.023 | 0.513 | 0.509 | 0.013 | 0.080 | 0.004 | 1.29E-09 | 37.036 | 521893 | 5 | 112113735 | APC | Intron Variant |
| rs4460001 | A | C | A | C | -0.019 | 0.004 | 0.432 | 0.358 | 0.013 | 0.774 | 0.004 | 4.45E-08 | 30.407 | 525491 | 4 | 130275243 | none | none |
| rs6457816 | T | C | T | C | -0.041 | 0.014 | 0.934 | 0.929 | 0.026 | 0.583 | 0.007 | 3.97E-09 | 34.979 | 525488 | 6 | 35362848 | PPARD | Intron Variant |
| rs7430216 | T | C | T | C | 0.025 | -0.010 | 0.223 | 0.189 | 0.015 | 0.516 | 0.004 | 2.50E-09 | 35.431 | 523040 | 3 | 75201030 | NIPA2P2 | Intron Variant |
| rs7615206 | T | C | T | C | -0.035 | 0.013 | 0.570 | 0.552 | 0.013 | 0.302 | 0.004 | 1.50E-22 | 97.163 | 521894 | 3 | 49937505 | MST1R | Intron Variant |
| rs7821826 | T | C | T | C | 0.021 | -0.015 | 0.493 | 0.492 | 0.013 | 0.234 | 0.004 | 3.25E-09 | 34.979 | 524166 | 8 | 10769439 | XKR6 | Intron Variant |
| rs7969719 | T | C | T | C | 0.027 | -0.022 | 0.313 | 0.292 | 0.014 | 0.114 | 0.004 | 4.46E-13 | 53.646 | 525490 | 12 | 109883577 | MYO1H | Intron Variant |

| **Table S9. Summary information on PA SNPs used as genetic instruments for the Mendelian randomization analyses with childhood IQ** | | | | | | | | | | | | | | | | | | |
| --- | --- | --- | --- | --- | --- | --- | --- | --- | --- | --- | --- | --- | --- | --- | --- | --- | --- | --- |
| **SNP** | **effect_allele.exposure** | **other_allele.exposure** | **effect_allele.outcome** | **other_allele.outcome** | **beta.**  **exposure** | **beta.**  **outcome** | **eaf.**  **exposure** | **eaf.**  **outcome** | **se.**  **outcome** | **pval.**  **outcome** | **se.**  **exposure** | **pval.**  **exposure** | **F-statistics** | **SampleSize** | **Chromosome** | **base pair location(GRCh37/hg19)** | **Gene** | **Functional anotation** |
| rs10210242 | A | G | A | G | 0.016 | -0.006 | 0.499 | 0.517 | 0.013 | 0.640 | 0.003 | 3.630E-07 | 25.000 | 600640 | 2 | 117322280 | none | none |
| rs10269727 | A | G | A | G | 0.029 | 0.024 | 0.934 | 0.900 | 0.026 | 0.359 | 0.006 | 4.610E-06 | 20.959 | 592095 | 7 | 122388255 | CADPS2 | Intron Variant |
| rs1029420 | T | C | T | C | -0.016 | -0.021 | 0.391 | 0.333 | 0.013 | 0.121 | 0.003 | 2.240E-06 | 21.869 | 592699 | 15 | 91441086 | none | none |
| rs10821761 | T | C | T | C | 0.018 | -0.004 | 0.388 | 0.366 | 0.013 | 0.781 | 0.003 | 6.710E-08 | 29.907 | 604256 | 10 | 62182269 | ANK3 | Intron Variant |
| rs11603071 | T | C | T | C | -0.016 | -0.007 | 0.616 | 0.625 | 0.013 | 0.583 | 0.003 | 1.110E-06 | 24.071 | 606024 | 11 | 16408248 | SOX6 | Intron Variant |
| rs11690624 | A | G | A | G | -0.022 | -0.014 | 0.128 | 0.142 | 0.020 | 0.482 | 0.005 | 4.310E-06 | 21.007 | 604254 | 2 | 184952225 | none | none |
| rs11951097 | A | G | A | G | 0.035 | -0.025 | 0.050 | 0.033 | 0.030 | 0.396 | 0.007 | 2.730E-06 | 21.988 | 589251 | 5 | 153237781 | none | none |
| rs12347361 | C | G | C | G | -0.015 | -0.027 | 0.648 | 0.725 | 0.014 | 0.045 | 0.003 | 3.090E-06 | 21.496 | 606819 | 9 | 1634202 | none | none |
| rs13262776 | T | G | T | G | -0.036 | 0.017 | 0.057 | 0.075 | 0.028 | 0.544 | 0.007 | 2.570E-07 | 26.470 | 604249 | 8 | 3187408 | CSMD1 | Intron Variant |
| rs149352 | A | G | A | G | -0.017 | -0.002 | 0.342 | 0.308 | 0.013 | 0.901 | 0.003 | 6.010E-07 | 25.304 | 602352 | 5 | 94198611 | MCTP1 | Intron Variant |
| rs1531519 | T | C | T | C | 0.017 | 0.011 | 0.380 | 0.383 | 0.013 | 0.384 | 0.003 | 3.730E-07 | 25.304 | 604221 | 18 | 53480206 | LOC105372130 | Intron Variant |
| rs2012741 | A | C | A | C | 0.017 | -0.011 | 0.606 | 0.625 | 0.013 | 0.416 | 0.003 | 1.700E-07 | 27.892 | 601795 | 4 | 93459947 | GRID2 | Intron Variant |
| rs2074117 | T | G | T | G | 0.017 | 0.023 | 0.352 | 0.297 | 0.014 | 0.095 | 0.003 | 1.970E-07 | 27.802 | 592523 | 7 | 111395390 | DOCK4 | Intron Variant |
| rs2281767 | T | C | T | C | -0.016 | -0.039 | 0.487 | 0.517 | 0.013 | 0.003 | 0.003 | 2.800E-07 | 25.946 | 603227 | 13 | 99099338 | FARP1 | Intron Variant |
| rs2675124 | A | G | A | G | -0.020 | -0.009 | 0.204 | 0.183 | 0.016 | 0.588 | 0.004 | 2.450E-07 | 27.093 | 604193 | 2 | 236631040 | AGAP1 | Intron Variant |
| rs3109891 | T | C | T | C | 0.017 | 0.011 | 0.597 | 0.585 | 0.013 | 0.384 | 0.003 | 8.960E-08 | 28.891 | 602220 | 15 | 50874964 | TRPM7 | Intron Variant |
| rs3799000 | T | C | T | C | -0.017 | -0.010 | 0.472 | 0.492 | 0.013 | 0.454 | 0.003 | 1.150E-07 | 27.235 | 604254 | 6 | 69715114 | ADGRB3 | Intron Variant |
| rs4743347 | T | C | T | C | 0.018 | -0.004 | 0.692 | 0.725 | 0.014 | 0.766 | 0.003 | 2.770E-07 | 26.796 | 606819 | 9 | 102175840 | LOC107987011 | Intron Variant |
| rs499878 | T | C | T | C | 0.016 | -0.001 | 0.660 | 0.702 | 0.014 | 0.930 | 0.003 | 3.630E-06 | 20.783 | 604242 | 11 | 114067550 | ZBTB16 | Intron Variant |
| rs6663 | A | G | A | G | -0.019 | -0.028 | 0.237 | 0.217 | 0.015 | 0.060 | 0.004 | 3.720E-07 | 25.817 | 602217 | 12 | 109886603 | KCTD10 | Non Coding Transcript Variant |
| rs716117 | A | G | A | G | 0.015 | -0.012 | 0.430 | 0.542 | 0.013 | 0.357 | 0.003 | 1.320E-06 | 23.160 | 604249 | 21 | 24297622 | none | none |
| rs7233417 | A | G | A | G | -0.016 | -0.009 | 0.353 | 0.408 | 0.013 | 0.522 | 0.003 | 1.050E-06 | 23.508 | 604127 | 18 | 35200180 | none | none |
| rs7422036 | A | G | A | G | -0.020 | -0.036 | 0.847 | 0.867 | 0.017 | 0.040 | 0.004 | 4.280E-06 | 21.286 | 604078 | 2 | 50506284 | NRXN1 | Intron Variant |
| rs7584099 | A | G | A | G | 0.015 | 0.003 | 0.528 | 0.508 | 0.013 | 0.800 | 0.003 | 3.590E-06 | 21.103 | 599258 | 2 | 148478336 | ACVR2A | Intron Variant |
| rs7678766 | T | G | T | G | 0.020 | -0.002 | 0.829 | 0.833 | 0.017 | 0.896 | 0.004 | 3.510E-06 | 21.556 | 600638 | 4 | 68011953 | none | none |
| rs7822348 | C | G | C | G | -0.018 | -0.001 | 0.735 | 0.775 | 0.015 | 0.949 | 0.004 | 5.490E-07 | 24.723 | 600637 | 8 | 135542483 | ZFAT | Intron Variant |
| rs9885024 | A | G | A | G | -0.016 | 0.003 | 0.313 | 0.322 | 0.014 | 0.840 | 0.003 | 2.390E-06 | 22.145 | 604255 | 5 | 124683432 | LOC101927421 | Intron Variant |
| rs9903845 | A | C | A | C | -0.020 | -0.010 | 0.310 | 0.356 | 0.014 | 0.474 | 0.003 | 6.050E-09 | 34.602 | 604232 | 17 | 50291181 | CA10 | Intron Variant |

| **Table S10. Reverse Mendelian randomization analysis results.** | | | | | | | | | | | | | | | | | | | | | | | | |
| --- | --- | --- | --- | --- | --- | --- | --- | --- | --- | --- | --- | --- | --- | --- | --- | --- | --- | --- | --- | --- | --- | --- | --- | --- |
| Note: Causal effects estimated using 5 two-sample Mendelian randomization methods (Inverse-variance weighted, MR-Egger, weighted median, weighted mode, and MR-RAPS). All statistical tests were two-sided. *P-*value < 1.25E-2 after Bonferroni correction was considered significant. | | | | | | | | | | | | | | | | | | | | | | | | |
| **Exposure** | **Outcome** | ***P-*value of MR-PRESSO global test** | **Cochran’s Q derived *P-*value** | ***P-*value of MR-Egger intercept** | **Inverse-variance weighted** | | | | **MR-Egger** | | | | **Weighted median** | | | | **weighted mode** | | | | **MR-RAPS** | | | |
|  |  |  |  |  | **Estimate (β)** | **95% lower CI** | **95% upper CI** | ***P-*value** | **Estimate (β)** | **95% lower CI** | **95% upper CI** | ***P-*value** | **Estimate (β)** | **95% lower CI** | **95% upper CI** | ***P-*value** | **Estimate (β)** | **95% lower CI** | **95% upper CI** | ***P-*value** | **Estimate (β)** | **95% lower CI** | **95% upper CI** | ***P-*value** |
| childhood IQ | LST | 0.824 | 0.017 | 0.214 | -0.039 | -0.171 | 0.094 | 0.568 | -0.259 | -0.423 | -0.094 | 0.200 | -0.034 | -0.142 | 0.074 | 0.538 | -0.039 | -0.185 | 0.107 | 0.652 | -0.044 | -0.111 | 0.022 | 0.194 |
| childhood IQ | PA | 0.207 | 0.295 | 0.599 | 0.030 | -0.035 | 0.095 | 0.364 | 0.093 | -0.091 | 0.276 | 0.504 | 0.025 | -0.055 | 0.106 | 0.536 | 0.018 | -0.083 | 0.120 | 0.757 | 0.031 | -0.031 | 0.093 | 0.326 |

| **Table S11. Summary information on childhood IQ SNPs used as genetic instruments for the Mendelian randomization analyses with PA and LST** | | | | | | | | | | | | | | | | | |
| --- | --- | --- | --- | --- | --- | --- | --- | --- | --- | --- | --- | --- | --- | --- | --- | --- | --- |
| **SNP** | **effect_allele.**  **exposure** | **other_allele.**  **exposure** | **effect_allele.**  **outcome** | **other_allele.**  **outcome** | **beta.**  **exposure** | **beta.**  **outcome** | **eaf.**  **exposure** | **eaf.**  **outcome** | **se.**  **outcome** | **pval.**  **outcome** | **se.**  **exposure** | **pval.**  **exposure** | **F-statistics** | **Chromosome** | **base pair location(GRCh37/hg19)** | **Gene** | **Functional anotation** |
| rs12456203 | T | C | T | C | 0.091 | -0.004 | 0.138 | 0.120 | 0.005 | 0.443 | 0.020 | 3.87E-06 | 21.385 | 18 | 22414326 | KCTD1 | Intron Variant |
| rs17086144 | A | T | A | T | 0.198 | -0.030 | 0.025 | 0.025 | 0.011 | 0.008 | 0.043 | 4.00E-06 | 21.267 | 4 | 66070815 | EPHA5 | Intron Variant |
| rs716580 | A | G | A | G | 0.061 | 0.005 | 0.583 | 0.565 | 0.004 | 0.167 | 0.013 | 3.61E-06 | 21.312 | 9 | 22326434 | none | none |

| **Table S12. Summary information on LST SNPs used as genetic instruments for the Mendelian randomization analyses with ICV** | | | | | | | | | | | | | | | | | | |
| --- | --- | --- | --- | --- | --- | --- | --- | --- | --- | --- | --- | --- | --- | --- | --- | --- | --- | --- |
| **SNP** | **effect_allele.exposure** | **other_allele.exposure** | **effect_allele.outcome** | **other_allele.outcome** | **beta.exposure** | **beta.outcome** | **eaf.exposure** | **eaf.outcome** | **se.outcome** | **pval.outcome** | **se.exposure** | **pval.exposure** | **samplesize.outcome** | **F-statistics** | **Chromosome** | **base pair location(GRCh37/hg19)** | **Gene** | **Functional anotation** |
| rs10041724 | T | C | T | C | 0.025 | -0.007 | 0.807 | 0.800 | 0.017 | 0.685 | 0.004 | 1.510E-08 | 11373 | 32.025 | 5 | 124273520 | LINC02240 | Intron Variant |
| rs10059100 | A | G | A | G | -0.022 | 0.011 | 0.607 | 0.628 | 0.014 | 0.439 | 0.004 | 4.680E-08 | 11373 | 30.391 | 5 | 120100784 | PRR16 | Intron Variant |
| rs1017550 | A | G | A | G | 0.020 | -0.004 | 0.586 | 0.628 | 0.014 | 0.784 | 0.004 | 2.850E-08 | 11373 | 31.360 | 10 | 63587683 | LINC02625 | Intron Variant |
| rs10222987 | A | G | A | G | -0.021 | -0.007 | 0.636 | 0.658 | 0.014 | 0.623 | 0.004 | 9.020E-09 | 11373 | 33.704 | 4 | 185946130 | none | none |
| rs10400776 | A | C | A | C | -0.026 | 0.007 | 0.259 | 0.244 | 0.015 | 0.641 | 0.004 | 3.450E-09 | 11373 | 34.649 | 14 | 97326366 | VRK1 | Intron Variant |
| rs10772643 | T | C | T | C | -0.039 | -0.016 | 0.892 | 0.869 | 0.020 | 0.421 | 0.006 | 5.880E-10 | 11373 | 38.560 | 12 | 13415288 | EMP1 | Intron Variant |
| rs10889193 | A | C | A | C | 0.024 | 0.007 | 0.555 | 0.835 | 0.018 | 0.689 | 0.004 | 4.940E-10 | 11265 | 38.822 | 1 | 61106174 | LOC124904193 | Non Coding Transcript Variant |
| rs11074658 | T | C | T | C | -0.024 | -0.003 | 0.591 | 0.611 | 0.014 | 0.838 | 0.004 | 9.210E-10 | 11373 | 37.555 | 16 | 10308335 | LOC107984900 | 500B Downstream Variant |
| rs114590429 | A | C | A | C | 0.038 | -0.035 | 0.116 | 0.108 | 0.021 | 0.099 | 0.006 | 3.030E-10 | 11373 | 39.271 | 2 | 166176789 | SCN2A | Intron Variant |
| rs11587591 | A | G | A | G | 0.022 | -0.024 | 0.734 | 0.745 | 0.015 | 0.114 | 0.004 | 2.230E-08 | 11373 | 31.533 | 1 | 209762875 | CAMK1G | Intron Variant |
| rs1188887 | T | C | T | C | 0.026 | -0.009 | 0.667 | 0.616 | 0.014 | 0.505 | 0.004 | 2.470E-10 | 11373 | 39.598 | 6 | 139257866 | REPS1 | Intron Variant |
| rs12062845 | A | C | A | C | 0.028 | -0.018 | 0.216 | 0.211 | 0.016 | 0.272 | 0.004 | 2.410E-11 | 11373 | 44.762 | 1 | 98342685 | DPYD | Intron Variant |
| rs12206846 | A | G | A | G | 0.020 | 0.021 | 0.405 | 0.420 | 0.013 | 0.111 | 0.004 | 2.100E-08 | 11373 | 30.556 | 6 | 108238917 | SEC63 | Intron Variant |
| rs12214364 | T | G | T | G | -0.020 | 0.001 | 0.587 | 0.624 | 0.014 | 0.947 | 0.004 | 4.870E-08 | 11373 | 29.945 | 6 | 67556372 | none | none |
| rs12324720 | A | G | A | G | -0.027 | 0.045 | 0.176 | 0.207 | 0.016 | 0.006 | 0.005 | 3.530E-09 | 11373 | 34.707 | 15 | 64092140 | HERC1 | Intron Variant |
| rs12425850 | T | C | T | C | -0.024 | 0.019 | 0.286 | 0.296 | 0.015 | 0.197 | 0.004 | 1.370E-09 | 11373 | 36.618 | 12 | 123501972 | PITPNM2 | Intron Variant |
| rs12463321 | A | G | A | G | -0.032 | 0.024 | 0.148 | 0.149 | 0.019 | 0.197 | 0.006 | 6.990E-09 | 11373 | 33.220 | 19 | 37651855 | ZNF585A | Intron Variant |
| rs12617870 | T | G | T | G | 0.026 | 0.001 | 0.538 | 0.533 | 0.013 | 0.966 | 0.004 | 6.620E-14 | 11373 | 56.464 | 2 | 193746283 | PCGEM1 | Intron Variant |
| rs12678836 | A | C | A | C | 0.023 | 0.003 | 0.424 | 0.396 | 0.014 | 0.814 | 0.004 | 5.370E-11 | 11373 | 43.938 | 8 | 92690148 | RN7SKP231 | Intron Variant |
| rs13017586 | A | G | A | G | -0.040 | 0.008 | 0.849 | 0.850 | 0.019 | 0.652 | 0.005 | 8.250E-14 | 11373 | 55.144 | 2 | 147847198 | none | none |
| rs13089152 | T | C | T | C | -0.023 | 0.011 | 0.669 | 0.674 | 0.014 | 0.426 | 0.004 | 1.530E-08 | 11373 | 32.573 | 3 | 84765574 | LINC00971 | Intron Variant |
| rs13301354 | T | C | T | C | -0.021 | 0.000 | 0.377 | 0.365 | 0.014 | 0.978 | 0.004 | 1.220E-08 | 11265 | 32.521 | 9 | 139924637 | LINC02908、FUT7、ABCA2 | Intron Variant、3 Prime UTR Variant、2KB Upstream |
| rs16896229 | T | C | T | C | 0.030 | -0.032 | 0.136 | 0.107 | 0.021 | 0.131 | 0.005 | 3.800E-09 | 11373 | 34.602 | 4 | 18002583 | LCORL | Intron Variant |
| rs17621391 | T | C | T | C | 0.024 | -0.007 | 0.735 | 0.728 | 0.015 | 0.645 | 0.004 | 2.110E-09 | 11373 | 35.127 | 7 | 140176596 | MKRN1 | Intron Variant |
| rs1802669 | A | G | A | G | 0.038 | -0.024 | 0.341 | 0.355 | 0.014 | 0.085 | 0.004 | 2.390E-21 | 11373 | 87.719 | 10 | 21827796 | MLLT10 | Synonymous Variant |
| rs1860337 | T | C | T | C | -0.025 | -0.009 | 0.595 | 0.545 | 0.013 | 0.493 | 0.004 | 9.080E-11 | 11373 | 42.083 | 17 | 60851559 | MARCHF10 | Intron Variant |
| rs1947066 | A | G | A | G | 0.030 | 0.007 | 0.803 | 0.822 | 0.017 | 0.702 | 0.004 | 8.540E-12 | 11373 | 45.870 | 5 | 161101615 | GABRA6 | Intron Variant |
| rs197439 | A | G | A | G | -0.026 | 0.022 | 0.601 | 0.567 | 0.013 | 0.104 | 0.004 | 3.290E-11 | 11373 | 44.103 | 1 | 112280990 | INKA2、INKA2-AS1 | Intron Variant、2KB Upstream Variant |
| rs1999065 | T | C | T | C | 0.025 | -0.022 | 0.338 | 0.343 | 0.014 | 0.122 | 0.004 | 1.240E-11 | 11373 | 45.289 | 9 | 120514574 | TLR4 | Intron Variant |
| rs249960 | A | G | A | G | 0.030 | 0.002 | 0.818 | 0.810 | 0.017 | 0.902 | 0.005 | 2.430E-09 | 11221 | 35.522 | 5 | 96164771 | ERAP1 | Intron Variant |
| rs2738284 | A | G | A | G | -0.022 | 0.031 | 0.329 | 0.380 | 0.014 | 0.022 | 0.004 | 7.050E-09 | 11373 | 33.766 | 2 | 217311609 | SMARCAL1 | Intron Variant |
| rs2783992 | T | C | T | C | -0.024 | -0.016 | 0.539 | 0.596 | 0.014 | 0.233 | 0.004 | 3.360E-10 | 11373 | 40.557 | 9 | 1722044 | LOC105375951 | Intron Variant |
| rs34864022 | A | G | A | G | -0.048 | 0.027 | 0.934 | 0.938 | 0.027 | 0.319 | 0.008 | 4.710E-10 | 11373 | 38.345 | 9 | 22609110 | DMRTA1 | Intron Variant |
| rs36079846 | T | C | T | C | -0.024 | 0.028 | 0.521 | 0.546 | 0.013 | 0.039 | 0.004 | 4.520E-10 | 11373 | 39.557 | 2 | 215367159 | VWC2L | Intron Variant |
| rs364789 | A | G | A | G | 0.028 | 0.004 | 0.757 | 0.786 | 0.016 | 0.807 | 0.004 | 2.790E-10 | 11373 | 39.063 | 5 | 77387439 | AP3B1 | Intron Variant |
| rs3781412 | A | G | A | G | -0.021 | -0.023 | 0.601 | 0.594 | 0.013 | 0.093 | 0.004 | 8.380E-09 | 11373 | 33.383 | 10 | 126715154 | CTBP2 | Missense Variant |
| rs396321 | T | C | T | C | -0.021 | -0.034 | 0.513 | 0.492 | 0.013 | 0.011 | 0.004 | 1.290E-09 | 11373 | 37.036 | 5 | 112113735 | APC | Intron Variant |
| rs421151 | A | G | A | G | -0.036 | 0.005 | 0.916 | 0.901 | 0.022 | 0.811 | 0.006 | 1.090E-08 | 11373 | 32.653 | 8 | 73462574 | KCNB2 | Intron Variant |
| rs4416502 | A | G | A | G | 0.029 | -0.004 | 0.200 | 0.232 | 0.016 | 0.786 | 0.005 | 1.380E-09 | 11373 | 36.754 | 4 | 77030872 | ART3 | Intron Variant |
| rs4460001 | A | C | A | C | -0.019 | 0.025 | 0.432 | 0.404 | 0.014 | 0.060 | 0.004 | 4.450E-08 | 11373 | 30.407 | 4 | 130275243 | none | none |
| rs4483592 | T | C | T | C | 0.036 | 0.003 | 0.163 | 0.179 | 0.017 | 0.870 | 0.005 | 3.970E-12 | 11373 | 47.929 | 11 | 65990439 | PACS1 | Intron Variant |
| rs469565 | T | C | T | C | -0.026 | 0.021 | 0.774 | 0.734 | 0.015 | 0.161 | 0.005 | 1.510E-08 | 11032 | 31.702 | 22 | 29952437 | NIPSNAP1 | Intron Variant |
| rs558134 | T | C | T | C | -0.023 | -0.013 | 0.384 | 0.429 | 0.015 | 0.385 | 0.004 | 5.050E-10 | 9487 | 39.063 | 6 | 12693454 | PHACTR1 | Intron Variant |
| rs56151256 | A | C | A | C | 0.029 | -0.020 | 0.750 | 0.785 | 0.016 | 0.226 | 0.004 | 1.170E-10 | 11373 | 41.955 | 15 | 78024806 | LINGO1 | Intron Variant |
| rs57092155 | T | C | T | C | -0.027 | 0.011 | 0.783 | 0.763 | 0.016 | 0.484 | 0.005 | 8.800E-09 | 11373 | 32.514 | 7 | 53856368 | LINC01446 | Intron Variant |
| rs58541850 | A | G | A | G | 0.052 | -0.018 | 0.059 | 0.062 | 0.039 | 0.642 | 0.008 | 1.720E-10 | 5732 | 40.524 | 6 | 166165563 | PDE10A | Intron Variant |
| rs6010651 | A | C | A | C | 0.024 | -0.029 | 0.620 | 0.615 | 0.014 | 0.033 | 0.004 | 3.340E-09 | 11373 | 34.516 | 20 | 62418243 | ZBTB46 | Intron Variant |
| rs6073637 | A | C | A | C | -0.023 | 0.003 | 0.524 | 0.495 | 0.013 | 0.805 | 0.004 | 4.590E-09 | 11373 | 35.059 | 20 | 43714051 | none | none |
| rs62134209 | A | G | A | G | 0.054 | 0.003 | 0.953 | 0.950 | 0.030 | 0.916 | 0.009 | 3.280E-09 | 11373 | 34.953 | 2 | 45093457 | none | none |
| rs62151809 | T | C | T | C | 0.023 | -0.007 | 0.453 | 0.495 | 0.013 | 0.618 | 0.004 | 3.900E-09 | 11373 | 34.178 | 2 | 104433256 | LOC105373520 | Intron Variant |
| rs6457816 | T | C | T | C | -0.041 | -0.009 | 0.934 | 0.942 | 0.028 | 0.764 | 0.007 | 3.970E-09 | 11373 | 34.979 | 6 | 35362848 | PPARD | Intron Variant |
| rs6556840 | A | G | A | G | 0.020 | -0.019 | 0.337 | 0.329 | 0.014 | 0.189 | 0.004 | 4.320E-08 | 11373 | 30.399 | 5 | 93463902 | LOC105379087 | Intron Variant |
| rs657412 | T | G | T | G | -0.033 | -0.001 | 0.105 | 0.079 | 0.025 | 0.977 | 0.006 | 1.400E-08 | 11373 | 32.372 | 13 | 99047250 | FARP1 | Intron Variant |
| rs6674314 | A | G | A | G | 0.027 | -0.047 | 0.813 | 0.811 | 0.017 | 0.006 | 0.005 | 2.300E-08 | 11373 | 31.269 | 1 | 243920895 | AKT3 | Intron Variant |
| rs6685030 | A | G | A | G | -0.022 | -0.007 | 0.480 | 0.451 | 0.013 | 0.616 | 0.004 | 5.270E-10 | 11373 | 38.440 | 1 | 171805284 | DNM3 | Intron Variant |
| rs6727997 | A | G | A | G | -0.021 | 0.000 | 0.345 | 0.336 | 0.014 | 0.984 | 0.004 | 2.040E-08 | 11373 | 31.603 | 2 | 146346285 | none | none |
| rs68049022 | T | C | T | C | 0.031 | -0.002 | 0.798 | 0.817 | 0.017 | 0.918 | 0.005 | 6.180E-11 | 11373 | 42.793 | 10 | 66407019 | LOC124902439 | Intron Variant |
| rs72671494 | T | C | T | C | -0.035 | -0.010 | 0.860 | 0.880 | 0.020 | 0.630 | 0.006 | 5.720E-10 | 11373 | 38.840 | 8 | 93195457 | none | none |
| rs7430216 | T | C | T | C | 0.025 | -0.009 | 0.223 | 0.212 | 0.016 | 0.587 | 0.004 | 2.500E-09 | 11373 | 35.431 | 3 | 75201030 | NIPA2P2 | Intron Variant |
| rs743699 | A | G | A | G | -0.027 | 0.016 | 0.743 | 0.748 | 0.015 | 0.301 | 0.004 | 1.180E-09 | 11373 | 36.823 | 4 | 3305116 | RGS12 | Intron Variant |
| rs7615206 | T | C | T | C | -0.035 | 0.027 | 0.570 | 0.592 | 0.013 | 0.044 | 0.004 | 1.500E-22 | 11373 | 97.163 | 3 | 49937505 | MST1R | Intron Variant |
| rs7616518 | A | G | A | G | 0.020 | 0.022 | 0.450 | 0.480 | 0.013 | 0.100 | 0.004 | 2.600E-08 | 11373 | 31.360 | 3 | 83530809 | none | none |
| rs7627290 | A | G | A | G | -0.023 | 0.015 | 0.541 | 0.549 | 0.013 | 0.272 | 0.004 | 2.360E-09 | 11373 | 35.083 | 3 | 165711001 | none | none |
| rs7821826 | T | C | T | C | 0.021 | 0.001 | 0.493 | 0.480 | 0.013 | 0.958 | 0.004 | 3.250E-09 | 11373 | 34.979 | 8 | 10769439 | XKR6 | Intron Variant |
| rs78394231 | T | C | T | C | -0.038 | 0.038 | 0.902 | 0.902 | 0.022 | 0.089 | 0.007 | 3.530E-09 | 11373 | 34.358 | 6 | 107649123 | PDSS2 | Intron Variant |
| rs78451709 | T | C | T | C | -0.028 | -0.010 | 0.795 | 0.788 | 0.016 | 0.558 | 0.005 | 9.660E-09 | 11373 | 33.303 | 18 | 41515058 | LOC105372088 | 2KB Upstream Variant |
| rs7969719 | T | C | T | C | 0.027 | 0.007 | 0.313 | 0.311 | 0.014 | 0.643 | 0.004 | 4.460E-13 | 11373 | 53.646 | 12 | 109883577 | MYO1H | Intron Variant |
| rs841020 | T | C | T | C | 0.027 | 0.032 | 0.192 | 0.218 | 0.016 | 0.048 | 0.004 | 1.150E-09 | 11373 | 37.377 | 10 | 125409953 | GPR26 | Intron Variant |
| rs892087 | T | C | T | C | -0.028 | -0.024 | 0.634 | 0.588 | 0.013 | 0.080 | 0.004 | 9.980E-13 | 11373 | 50.410 | 19 | 10794793 | ILF3 | Intron Variant |
| rs9867121 | A | C | A | C | -0.032 | 0.005 | 0.183 | 0.186 | 0.017 | 0.776 | 0.005 | 2.020E-10 | 11373 | 40.196 | 3 | 114631548 | ZBTB20 | Intron Variant |

| **Table S13. Summary information on PA SNPs used as genetic instruments for the Mendelian randomization analyses with ICV** | | | | | | | | | | | | | | | | | | | |
| --- | --- | --- | --- | --- | --- | --- | --- | --- | --- | --- | --- | --- | --- | --- | --- | --- | --- | --- | --- |
| **SNP** | **effect_allele.exposure** | **other_allele.exposure** | **effect_allele.outcome** | **other_allele.outcome** | **beta.exposure** | **beta.outcome** | **eaf.exposure** | **eaf.outcome** | **se.outcome** | **pval.outcome** | **samplesize.outcome** | **se.exposure** | **pval.exposure** | **F-statistics** | **SampleSize** | **Chromosome** | **base pair location(GRCh37/hg19)** | **Gene** | **Functional anotation** |
| rs13201721 | T | C | T | C | 0.026 | -0.007 | 0.736 | 0.735 | 0.015 | 0.619 | 11373 | 0.004 | 1.83E-10 | 40.641 | 522597 | 6 | 141799534 | RPS3AP24 | Intron Variant |
| rs1691471 | T | C | T | C | 0.038 | 0.003 | 0.376 | 0.380 | 0.014 | 0.840 | 11373 | 0.004 | 1.73E-19 | 81.429 | 483768 | 3 | 85011013 | CADM2 | Intron Variant |
| rs4865512 | A | G | A | G | 0.024 | 0.002 | 0.612 | 0.649 | 0.014 | 0.882 | 11373 | 0.004 | 7.68E-09 | 32.653 | 483768 | 5 | 50661601 | ISL1-DT | Intron Variant |
| rs568546 | T | C | T | C | 0.024 | 0.018 | 0.521 | 0.536 | 0.013 | 0.171 | 11373 | 0.004 | 5.89E-09 | 33.414 | 483768 | 11 | 107321156 | CWF19L2 | Intron Variant |
| rs6427178 | A | G | A | G | 0.023 | -0.015 | 0.532 | 0.522 | 0.013 | 0.248 | 11373 | 0.004 | 1.71E-08 | 31.196 | 484633 | 1 | 169095082 | ATP1B1 | Intron Variant |
| rs9903845 | A | C | A | C | -0.020 | -0.006 | 0.310 | 0.285 | 0.015 | 0.698 | 11373 | 0.003 | 6.05E-09 | 34.602 | 604232 | 17 | 50291181 | CA10 | Intron Variant |

| **Table S14. Summary information on ICV SNPs used as genetic instruments for the Mendelian randomization analyses with childhood IQ** | | | | | | | | | | | | | | | | | | |
| --- | --- | --- | --- | --- | --- | --- | --- | --- | --- | --- | --- | --- | --- | --- | --- | --- | --- | --- |
| **SNP** | **effect_allele.exposure** | **other_allele.exposure** | **effect_allele.outcome** | **other_allele.outcome** | **beta.exposure** | **beta.outcome** | **eaf.exposure** | **eaf.outcome** | **se.outcome** | **pval.outcome** | **se.exposure** | **pval.exposure** | **F-statistics** | **SampleSize** | **Chromosome** | **base pair location(GRCh37/hg19)** | **Gene** | **Functional anotation** |
| rs1490384 | T | C | T | C | 0.062 | 0.024 | 0.492 | 0.433 | 0.013 | 0.063 | 0.013 | 2.640E-06 | 22.063 | 11373 | 1 | 111209920 | MIR588 | Tf binding site variant |
| rs17689882 | A | G | A | G | -0.095 | -0.020 | 0.230 | 0.200 | 0.015 | 0.188 | 0.016 | 2.870E-09 | 35.273 | 10944 | 17 | 43906828 | CRHR1\LINC02210-CRHR1 | Intron Variant |
| rs2022464 | A | C | A | C | -0.071 | -0.046 | 0.338 | 0.300 | 0.014 | 0.001 | 0.014 | 3.730E-07 | 25.831 | 11373 | 6 | 108945370 | FOXO3 | Intron Variant |
| rs2125644 | A | G | A | G | -0.164 | -0.042 | 0.036 | 0.033 | 0.039 | 0.284 | 0.036 | 4.600E-06 | 20.998 | 11373 | 12 | 46622346 | SLC38A1 | Intron Variant |
| rs744576 | C | T | C | T | 0.065 | 0.015 | 0.457 | 0.475 | 0.013 | 0.254 | 0.013 | 1.050E-06 | 23.838 | 11373 | 20 | 21030244 | none | none |

| **Table S15. Two-step Mendelian randomization analysis results.** | | | | | | | | | | | | | | | | | | | | | | | | |
| --- | --- | --- | --- | --- | --- | --- | --- | --- | --- | --- | --- | --- | --- | --- | --- | --- | --- | --- | --- | --- | --- | --- | --- | --- |
| Note: Causal effects estimated using 5 two-sample Mendelian randomization methods (inverse-variance weighted, MR-Egger, weighted median, weighted mode, MR-RAPS). All statistical tests were two-sided. P-value < 1.67E-2 after Bonferroni correction was considered significant. | | | | | | | | | | | | | | | | | | | | | | | | |
| **Exposure** | **Outcome** | P**-value of MR-PRESSO global test** | **Cochran’s Q derived** P-**value** | P**-value of MR-Egger intercept** | **Inverse-variance weighted** | | | | **MR-Egger** | | | | **Weighted median** | | | | **weighted mode** | | | | **MR-RAPS** | | | |
|  |  |  |  |  | **Estimate (β)** | **95% lower CI** | **95% upper CI** | P-**value** | **Estimate (β)** | **95% lower CI** | **95% upper CI** | P-**value** | **Estimate (β)** | **95% lower CI** | **95% upper CI** | P-**value** | **Estimate (β)** | **95% lower CI** | **95% upper CI** | P-**value** | **Estimate (β)** | **95% lower CI** | **95% upper CI** | P-**value** |
| LST | ICV | 0.059 | 0.057 | 0.308 | -0.227 | -0.381 | -0.072 | 0.004 | -0.597 | -1.322 | 0.127 | 0.111 | -0.193 | -0.391 | 0.006 | 0.057 | -0.048 | -0.551 | 0.456 | 0.854 | -0.234 | -0.374 | -0.095 | 0.001 |
| PA | ICV | 0.994 | 0.604 | 0.459 | 0.044 | -0.378 | 0.466 | 0.838 | 0.052 | -1.879 | 1.982 | 0.961 | 0.075 | -0.434 | 0.584 | 0.772 | 0.064 | -0.595 | 0.723 | 0.857 | 0.045 | -0.389 | 0.478 | 0.840 |
| ICV | childhood IQ | 0.379 | 0.463 | 0.528 | 0.334 | 0.162 | 0.505 | 0.000 | 0.096 | -0.580 | 0.773 | 0.799 | 0.238 | 0.009 | 0.468 | 0.042 | 0.223 | -0.065 | 0.510 | 0.204 | 0.343 | 0.153 | 0.533 | 0.000 |
